# Posicionamento sobre Exercícios Físicos na Gestação e no Pós-Parto – 2021

**DOI:** 10.36660/abc.20210408

**Published:** 2021-07-15

**Authors:** Milena dos Santos Barros Campos, Susimeire Buglia, Cléa Simone Sabino de Souza Colombo, Rica Dodo Delmar Buchler, Adriana Soares Xavier de Brito, Carolina Christianini Mizzaci, Roberta Helena Fernandes Feitosa, Danielle Batista Leite, Carlos Alberto Cordeiro Hossri, Lorena Christine Araújo de Albuquerque, Odilon Gariglio Alvarenga de Freitas, Gabriel Blacher Grossman, Luiz Eduardo Mastrocola

**Affiliations:** 1 Hospital São Lucas AracajuSE Brasil Hospital São Lucas, Rede D’Or, Aracaju, SE – Brasil; 2 Hospital Universitário de Sergipe AracajuSE Brasil Hospital Universitário de Sergipe, Aracaju, SE – Brasil; 3 Instituto Dante Pazzanese de Cardiologia São PauloSP Brasil Instituto Dante Pazzanese de Cardiologia, São Paulo, SP – Brasil; 4 Hospital do Coração São PauloSP Brasil Hospital do Coração (HCOR), São Paulo, SP – Brasil; 5 Faculdade de Medicina São Leopoldo Mandic CampinasSP Brasil Faculdade de Medicina São Leopoldo Mandic, Campinas, SP – Brasil; 6 Ergometria DASA São PauloSP Brasil Ergometria DASA, São Paulo, SP – Brasil; 7 Instituto Nacional de Cardiologia Rio de JaneiroRJ Brasil Instituto Nacional de Cardiologia, Rio de Janeiro, RJ – Brasil; 8 Hospital Copa Star – RDSL Rio de JaneiroRJ Brasil Hospital Copa Star – RDSL, Rio de Janeiro, RJ – Brasil; 9 Universidade Rio Verde GoiâniaGO Brasil Universidade Rio Verde (UniRV), Goiânia, GO – Brasil; 10 Secretaria Municipal de Saúde GoiâniaGO Brasil Secretaria Municipal de Saúde, Goiânia, GO – Brasil; 11 Real Hospital Português RecifePE Brasil Real Hospital Português, Recife, PE – Brasil; 12 Pronto Socorro Cardiológico de Pernambuco RecifePE Brasil Pronto Socorro Cardiológico de Pernambuco (PROCAPE), Recife, PE – Brasil; 13 Minascor Centro Médico Belo HorizonteMG Brasil Minascor Centro Médico, Belo Horizonte, MG – Brasil; 14 Hospital Moinhos de Vento Porto AlegreRS Brasil Hospital Moinhos de Vento, Porto Alegre, RS – Brasil; 15 Clínica Cardionuclear Porto AlegreRS Brasil Clínica Cardionuclear, Porto Alegre, RS – Brasil

**Table:** Posicionamento sobre Exercícios Físicos na Gestação e no Pós-Parto – 2021

O relatório abaixo lista as declarações de interesse conforme relatadas à SBC pelos especialistas durante o período de desenvolvimento deste posicionamento, 2020.	
Especialista	Tipo de relacionamento com a indústria
Adriana Soares Xavier de Brito	Nada a ser declarado
Carlos Alberto Cordeiro Hossri	Nada a ser declarado
Carolina Christianini Mizzaci	Nada a ser declarado
Cléa Simone Sabino de Souza Colombo	Nada a ser declarado
Danielle Batista Leite	Outros relacionamentos Participação societária de qualquer natureza e qualquer valor economicamente apreciável de empresas na área de saúde, de ensino ou em empresas concorrentes ou fornecedoras da SBC: – Curso de Ergometria: GEFE
Gabriel Blacher Grossman	Nada a ser declarado
Lorena Christine Araújo de Albuquerque	Nada a ser declarado
Luiz Eduardo Mastrocola	Nada a ser declarado
Milena dos Santos Barros Campos	Nada a ser declarado
Odilon Gariglio Alvarenga de Freitas	Nada a ser declarado
Rica Dodo Delmar Buchler	Nada a ser declarado
Roberta Helena Fernandes Feitosa	Nada a ser declarado
Susimeire Buglia	Nada a ser declarado

## 1. Introdução

O Departamento de Ergometria, Exercício, Cardiologia Nuclear e Reabilitação Cardiovascular (DERC) por meio da comissão DERC mulher (Saúde e Diagnóstico das Doenças Cardiovasculares nas Mulheres), Grupo de Estudos da Cardiologia do Esporte (GECESP) e Grupo de Estudos de Reabilitação Cardiopulmonar e Metabólica (GERCPM), elaborou este documento de acordo com as normas da Sociedade Brasileira de Cardiologia com a finalidade de orientar aos profissionais de saúde a prescrição dos exercícios físicos (EF) nos períodos gestacional e pós-parto, assim como fortalecer a relação entre os especialistas nas áreas relacionadas (clínicos, cardiologistas e obstetras em especial), incluindo o manejo dos EF nas gestantes atletas e nas portadoras de comorbidades. Ao final deste documento, serão abordadas as peculiaridades da prática dos EF no momento atual da pandemia pelo novo coronavírus.

Os EF apresentam benefícios comprovados na promoção de saúde materno-fetal, porém as mulheres, principalmente as que têm comorbidades, acabam reduzindo as atividades físicas ou permanecem sedentárias na gestação, com receio das complicações clínicas e obstétricas. Portanto, há a necessidade de ratificação das vantagens dos EF na melhora da capacidade funcional, na redução da fadiga, na diminuição do risco de depressão, na prevenção do ganho de peso excessivo, no auxílio ao controle dos distúrbios metabólicos e cardiovasculares, como, por exemplo, pré-eclâmpsia, distúrbios hipertensivos e diabetes melito desenvolvidos na gestação. Estes são hoje considerados fatores de risco emergentes ou específicos para o sexo e estão associados ao maior risco de doenças cardiovasculares (DCV).

As consultas planejadas ao obstetra representam ótima oportunidade de incentivo e conscientização de estilo de vida mais saudável. Em geral, as mulheres ativas durante a gestação mantêm os mesmos hábitos no período pós-parto, com benefícios estabelecidos na saúde cardiovascular a longo prazo. Nesta fase de pandemia da doença do novo coronavírus 2019 (COVID-19), observou-se aumento do sedentarismo, com a necessidade de intensificação das recomendações dos EF em gestantes, orientadas para os cuidados habituais de prevenção da contaminação e disseminação do vírus. As gestantes, principalmente na presença de comorbidades, devem ser encaminhadas ao especialista para contribuir no controle de fatores de risco para DCV e na avaliação e prescrição dos EF.

Tal posicionamento abordará a descrição dos benefícios e indicações dos EF na gestação e pós-parto, a padronização da avaliação clínica pré-participação, orientações na prescrição, nos cuidados e critérios de interrupção dos EF e as particularidades suscitadas na pandemia da COVID-19.

## 2. Exercícios Físicos na Gestação

### 2.1. Benefícios e Indicações

A gravidez provoca alterações hormonais e anatômicas que levam a hiperlordose lombar, afrouxamento dos ligamentos da cintura pélvica, retenção de líquido no tecido conjuntivo e aumento do peso corporal. Consequentemente, há sobrecarga da coluna vertebral e lombalgia como sintoma predominante em aproximadamente 60% das gestantes, interferindo negativamente em: qualidade do sono, disposição física, desempenho no trabalho, vida social, atividades domésticas e lazer.^1^ Há também tendência à redução da prática de atividade física durante a gravidez, principalmente no terceiro trimestre, o que agrava as implicações anatômicas da gestação. As recomendações e diretrizes de ginecologia e obstetrícia e da cardiologia orientam os EF na gravidez na promoção da saúde materna, fetal e neonatal.^2^^–^^4^ Os EF reduzem o percentual de massa gorda, aumentam a transferência de oxigênio (O_2_) e reduzem a difusão de dióxido de carbono (CO_2_) por meio da placenta, favorecendo o desenvolvimento fetal. Adicionalmente, a prática regular diminui em torno de 50% o risco de diabetes melito gestacional (DMG), em até 40% de pré-eclâmpsia (PE), hipertensão arterial gestacional (HG), ganho de peso excessivo e depressão.^5^^–^^8^

Na ausência de comordidades, as gestantes devem iniciar os EF assim que se sentirem dispostas, considerando-se que a fadiga e os sintomas indesejados do início da gravidez podem atrapalhar. Os EF orientados são vistos como seguros para a mãe e para o feto, sem o relato de aumento na frequência de anomalias congênitas, de parto prematuro e de baixo peso ao nascer.^9^^–^^12^ É importante que a paciente seja avaliada pelo obstetra e/ou cardiologista antes de iniciar os EF.

As indicações da realização dos EF na gravidez estão descritas na Tabela 1, conforme níveis de evidência e qualidade das recomendações, adotadas em diretriz internacional recentemente publicada.^3^ A força das recomendações foi estabelecida por um sistema denominado GRADE^13^ (*Grading of Recommendations Assessment, Development and Evaluation System*), classificadas como fortes ou fracas de acordo com: **(1)** balanço entre benefícios e prejuízo; **(2)** qualidade geral das evidências; **(3)** importância dos desfechos; **(4)** utilização de recursos – custo; **(5)** viabilidade; **(6)** aceitabilidade.

**Tabela 1:** Recomendações para a prática de exercícios durante a gravidez: qualidade e força das evidências para atividade física durante a gestação

Indicações	Recomendação	Qualidade das evidências
Todas as mulheres sem contraindicações devem ser fisicamente ativas durante todo o período gestacional (subgrupos a, b, c)	Forte	Moderada
Mulheres previamente inativas	Forte	Moderada
Mulheres com diabetes gestacional	Fraca	Baixa
IMC pré-gestacional ≥ 25 Kg/m^2^	Forte	Baixa
Gestantes devem acumular o mínimo de 150 min de exercícios de moderada intensidade/semana, pelo menos 3 dias/semana	Forte	Moderada
Gestantes devem incorporar atividades aeróbicas e de resistência variadas. Exercícios de alongamento e ioga podem ser adicionados	Forte	Alta
Treinamento para “assoalho pélvico” (exercícios de Kegel) diários (reduzir risco de incontinência urinária)	Fraca	Baixa

*Modificada de Mottola et al*.^3^

### 2.2. Adaptações Cardiovasculares e Respiratórias Fisiológicas em Repouso e ao Exercício

O conhecimento das adaptações fisiológicas cardiovasculares e respiratórias é de grande importância para a compreensão dos sintomas cardiorrespiratórios comuns na gestação e auxílio ao diagnóstico diferencial de sintomas relacionados à eventual descompensação de cardiopatias previamente estáveis, bem como na orientação de EF durante esse período.

**Repouso:** Na gravidez ocorre aumento do volume sanguíneo secundário ao aumento do plasma e das hemácias, levando à “anemia por efeito dilucional”, principalmente ao final do período gestacional. Mesmo em repouso, verifica-se aumento do volume sistólico (VS) e também da frequência cardíaca (FC), ocasionando elevação do débito cardíaco (DC).^14^^,^^15^ Também se observa modificação na excitabilidade cardíaca, propiciando maior ocorrência de extrassistolia. O efeito estrogênico promove a diminuição do tônus vascular, com queda da resistência vascular periférica (RVP) e consequente redução da pressão arterial (PA), em especial a diastólica. Apesar de a RVP permanecer baixa, as variações de fluidos que ocorrem próximo ao parto ocasionam labilidade da PA nessa fase e facilitam o aparecimento de edema de membros inferiores.^16^ Tais mudanças, além de habitualmente gerarem maior sensação de palpitação ao repouso e tontura pós-esforço, devem ser consideradas fatores predisponentes para a descompensação de cardiopatias preexistentes.^17^

**Figura 1:**
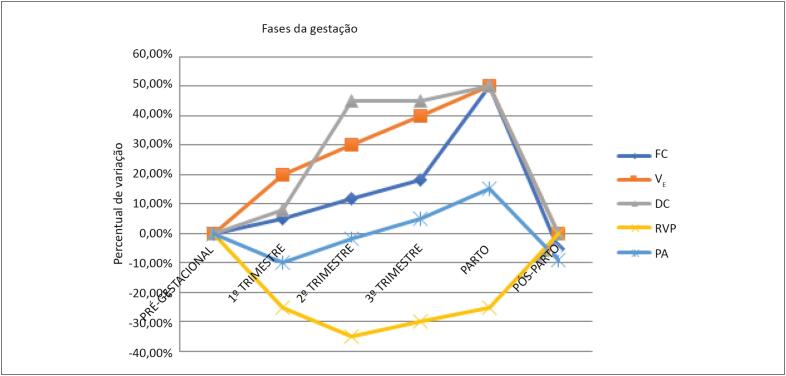
Modificações fisiológicas durante o período de gestação. DC: débito cardíaco; FC: frequência cardíaca; PA: pressão arterial; RVP: resistência vascular periférica; VE: ventilação-minuto.

O aumento dos níveis de oxigênio é mecanismo importante de adaptação materna para facilitar a transferência para o feto através da placenta. As demandas metabólicas aumentadas do feto, útero e do organismo materno resultam em aumento do consumo de oxigênio (VO_2_), da produção de gás carbônico (VCO_2_) e da taxa metabólica basal, causando hiperventilação, também influenciada pela progesterona. Há aumento significativo da ventilação-minuto (V_E_) e do volume corrente, com elevação da pressão parcial de O_2_ no sangue arterial e diminuição da pressão parcial de CO_2_. A capacidade funcional residual diminui, principalmente devido ao deslocamento do diafragma, o que aumenta a sensação de desconforto respiratório mesmo em repouso. Essas alterações explicam a “dispneia fisiológica” observada em cerca de 60% a 70% das gestantes saudáveis, comum próximo a 30ᵃ semana, bem como a redução da capacidade de se manter em apneia, o que limita atividades como mergulho e exercícios com maior componente anaeróbico, como corridas de velocidade.^18^ As adaptações fisiológicas durante o período de gestação estão descritas na Figura 1.

**Exercício:** Durante o exercício, as variáveis VS, DC, FC e V_E_ também sofrem incremento, com aumento da capacidade aeróbica já no primeiro trimestre da gestação e maior rendimento nesse período. Apesar de a FC da gestante ser maior ao repouso, ela não se eleva da mesma forma durante o exercício, e o VO_2_ não aumenta proporcionalmente ao incremento das cargas, principalmente quando o esforço inclui a sustentação do peso corporal. Dessa forma, ocorre limitação da capacidade funcional com a progressão da gestação. O aumento da V_E_ excede o aumento do VO_2_, mas a diferença arteriovenosa de O_2_ diminui, o que proporciona maior entrega de oxigênio para o feto; entretanto, em exercícios de maior intensidade, há risco de desvio de fluxo de sangue do útero para os músculos, o que pode ser prejudicial ao desenvolvimento fetal.^18^^,^^19^

Do ponto de vista anatômico, observa-se aumento das dimensões da cavidade do ventrículo esquerdo (VE), sem aumento da espessura de parede. Estudo com 105 grávidas demonstrou que 35% desenvolveram aumento de trabeculações em VE, 8% preenchendo critérios para diagnóstico de miocárdio não compactado. Tais alterações são relatadas em outras situações em que ocorre aumento de DC, como nas atletas, sendo reversíveis após o retorno às condições hemodinâmicas basais e não devem ser confundidas com alterações patológicas.^20^

### 2.3. Avaliação para a Prática de Exercícios

**Figura 2:**
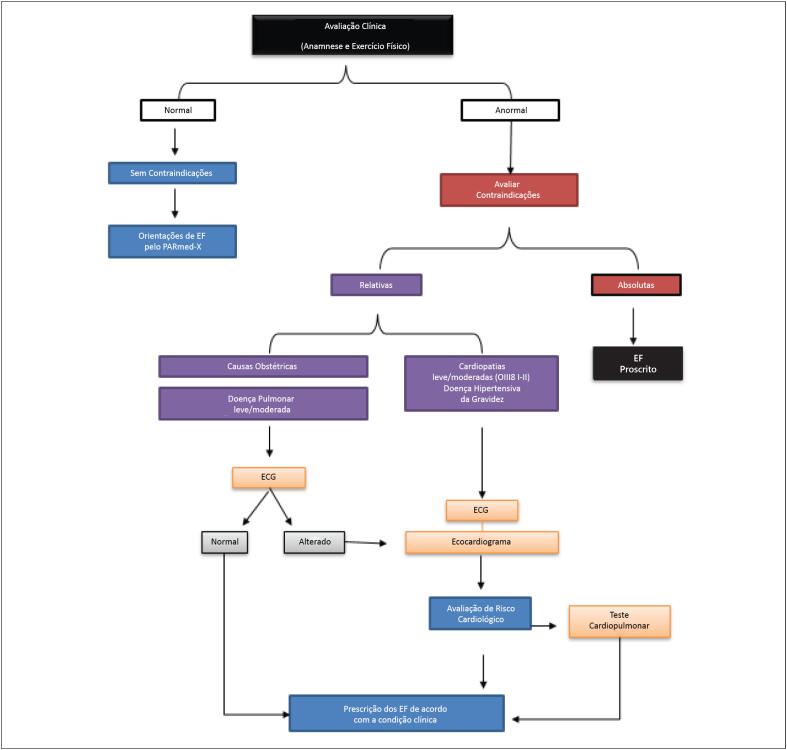
Avaliação da gestante para realização dos exercícios. ECG: eletrocardiograma; EF: exercícios físicos; OMS: Organização Mundial da Saúde.

Tanto a recomendação quanto o impedimento para a realização dos EF (Figura 2) durante a gestação geralmente são feitos pelo obstetra, após avaliação clínica e identificação da presença ou não das contraindicações, tais como presença de doenças preexistentes não controladas, complicações médicas ou obstétricas, dentre outras (Tabela 2).^3^ As mulheres com DCV devem ser acompanhadas também pelo cardiologista. Atualmente, a parceria da cardiologia com a ginecologia e obstetrícia permite a identificação precoce e a modificação dos fatores de risco nas mulheres para DCV.^21^

**Tabela 2:** Contraindicações para atividade física na gravidez

CAUSAS OBSTÉTRICAS	
ABSOLUTAS	RELATIVAS
Rotura da membrana	Antecedente de parto prematuro
Trabalho de parto prematuro	Perda recorrente de gestação
Placenta prévia após 28 semanas de gestação	Gravidez gemelar >28 semanas
Gestação múltipla (trigemelar ou superior)	
Sangramento vaginal persistente inexplicado	
Incontinência istmo-cervical	
Restrição de crescimento intrauterino	

CAUSAS CLÍNICAS	
ABSOLUTAS	RELATIVAS
Hipertensão arterial crônica (não controlada)/pré-eclâmpsia	Doença hipertensiva da gravidez
Doença cardiovascular grave (OMS III-IV)	Doença cardiovascular leve/moderada (OMS I-II)
Doença pulmonar restritiva	Doença pulmonar leve/moderada
Diabetes melito tipo I, doença da tireoide (não controladas)	Obesidade extrema, desnutrição ou transtorno alimentar Anemia sintomática

*OMS: Organização Mundial da Saúde*.

O PARmed-X (*Physical Activity Readiness Medical Examination*) foi desenvolvido pela Sociedade Canadense de Fisiologia do Exercício, desenhado para avaliação e orientação de exercícios na gravidez, muito bem aceito por diversas sociedades médicas no mundo por sua praticidade, e inclui a seguinte série de verificação: **a)** saúde pré-exercício (preenchida pela gestante); **b)** seção relacionada a contraindicações ao exercício; **c)** avaliação de saúde (preenchido pelo médico assistente) para ser usada pelo profissional de educação física; **d)** acrescenta adicionalmente instruções para a prescrição de exercícios aeróbicos e de condicionamento muscular, fornecendo assim recomendações para prescrição segura e individualizada, como também indicadores para o momento da sua interrupção.^22^

Após minuciosa anamnese e exame clínico, pode-se recomendar e orientar os EF na gravidez. Não existe a indicação de exames cardiológicos de rotina para todas as gestantes, tornando a necessidade específica de acordo com a condição clínica. O eletrocardiograma (ECG) não faz parte da rotina pré-natal. O método deve ser utilizado na investigação de cardiopatia, acompanhamento de gestantes com DCV prévia e avaliação de arritmias.^23^ Recomenda-se o ECG, além dessas indicações, nas pacientes que apresentarem contraindicações clínicas e obstétricas relativas aos EF (Tabela 2).

O ecocardiograma transtorácico é o método de imagem preferido quando se suspeita de DCV; trata-se de modalidade diagnóstica não invasiva, de fácil execução e amplamente disponível.^24^ Recomenda-se sua utilização nas portadoras de cardiopatia prévia, com sintomas sugestivos de cardiopatia ou ECG alterado.

A realização do teste de esforço (TE) máximo para avaliação funcional em gestantes não é recomendada. Se absolutamente necessário para avaliação cardiovascular e após exclusão das contraindicações, a paciente poderá realizar prova submáxima (até 85% da FC máxima prevista), considerada segura pelas Sociedades Brasileira e Europeia de Cardiologia. Entretanto, a carência de estudos não possibilita validar a sua indicação para a caracterização de doença isquêmica do coração. Neste contexto, o ecocardiograma sob estresse físico em bicicleta ergométrica pode melhorar a especificidade do diagnóstico da doença arterial coronariana, por agregar a imagem aos achados do TE submáximo, sendo contraindicado o uso do estresse farmacológico com dobutamina durante a gravidez.^24^^,^^25^

Em relação à prescrição de exercício, principalmente em pacientes com maior risco cardiovascular, o TE submáximo é mais eficiente quando associado à análise de gases expirados, ou seja, a realização do teste cardiopulmonar de exercício (TCPE), que possibilita a determinação dos índices de limitação funcional e limiares ventilatórios, tornando a prescrição mais precisa, segura e objetiva.^26^^,^^27^ Não existe boa correlação entre carga de esforço e FC para avaliação de gestantes,^28^ sendo o TCPE realizado nas cardiopatas grávidas em situações específicas (doença valvar e cardiopatias congênitas), após exclusão das contraindicações clínicas e obstétricas absolutas e em nível submáximo. Não é recomendado como exame de rotina.

A análise do condicionamento físico em grávidas pode ajudar a identificar as que desenvolveram ou estão em risco de desenvolver complicações cardiovasculares, como hipertensão arterial sistêmica (HAS) ou PE.O teste de caminhada de 6 minutos (TC6min) é avaliação validada da reserva cardiorrespiratória em adultos de alto risco e não gestantes, tanto em patologias crônicas quanto na avaliação perioperatória. É prova submáxima, segura, viável e aplicável em mulheres grávidas a termo, mas seu uso não é difundido. O estabelecimento de intervalos de referência para o TC6min em mulheres grávidas saudáveis permite a avaliação individual da capacidade funcional e pode ajudar a personalizar os programas de exercícios.^29^^,^^30^ Recomenda-se o TC6min na avaliação cardiorrespiratória de gestantes.

Sendo assim e, geralmente na presença de doenças preexistentes e contraindicações clínicas ou obstétricas relativas aos EF, impõem-se exames cardiológicos mais específicos antes da prática dos EF durante a gravidez.

#### 2.3.1. Estratificação de Risco da Gestante Cardiopata

As pacientes portadoras de DCV devem ser estratificadas quanto ao risco para liberação da prática de EF. O risco de complicações na gravidez depende do diagnóstico cardíaco subjacente, mas deve ser levada em consideração a associação de outras comorbidades. Portanto, as estimativas de risco devem ser individualizadas.

A capacidade funcional e a estabilidade clínica nas cardiopatias são fatores importantes a serem avaliados para análise do risco materno de complicações, mas, muitas vezes, é difícil a diferenciação de sinais e sintomas fisiológicos à gestação ou secundários à descompensação da cardiopatia, tais como edema, dispneia, palpitação e tontura. No entanto, devem ser valorizadas queixas como palpitações, piora da capacidade funcional, tosse seca noturna, ortopneia, dispneia paroxística noturna, hemoptise, dor precordial ao esforço e síncope.^24^

Nas gestantes com cardiopatia, é necessária abordagem mais detalhada por meio de exames específicos, como saturação de oxigênio, bioquímica sanguínea (níveis de peptídios natriuréticos), ecocardiograma (função ventricular, acometimento das válvulas, pressões intrapulmonares e diâmetros aórticos), TE ou TCPE submáximo (capacidade de exercício) e *holter* (arritmias).^25^

O risco da cardiopatia durante a evolução da gravidez pode ser estratificado usando a classificação modificada da Organização Mundial da Saúde (OMS) (Tabela 3).^31^^–^^33^ Mulheres com doença cardíaca têm risco aumentado de complicações obstétricas, incluindo parto prematuro, pré-eclâmpsia e hemorragia pós-parto. Complicações fetais também ocorrem em 18% a 30% das pacientes com cardiopatia, com mortalidade neonatal entre 1% e 4%. Os eventos maternos e fetais são altamente correlacionados.^34^^,^^35^

**Tabela 3:** Classificação modificada da Organização Mundial da Saúde considerando-se o risco cardiovascular materno

OMS modificada	I	II	II-III	III	IV
Diagnóstico	Pequeno ou leve: – Estenose pulmonar – *Ductus arteriosus* patente – Prolapso valvar mitral (lesões simples reparadas com sucesso) – Ectopias atriais ou ventriculares isoladas	– Defeito septal atrioventricular não operado – Tetralogia de Fallot corrigida – Arritmias supraventriculares – Síndrome de Turner sem dilatação aórtica	– Disfunção ventricular leve (FE >45%) – Cardiomiopatia hipertrófica – Doença valvar não considerada na classe OMS I ou IV (estenose mitral leve, estenose aórtica moderada) – Síndrome de Marfan sem dilatação aórtica – Coarctação corrigida – Defeito septal atrioventricular	– Disfunção ventricular moderada (FE 30% a 45%) – Cardiomiopatia periparto prévia sem disfunção ventricular residual – Válvula mecânica – Circulação de Fontan – Cardiopatia cianótica não corrigida – Outras doenças cardíacas complexas – Estenose mitral moderada – Estenose aórtica grave assintomática – Ventrículo direito sistêmico com função preservada ou disfunção leve – Dilatação aórtica moderada (40 a 45mm na síndrome de Marfan; 45 a 50mm na válvula aórtica bicúspide; ITA 20 a 25mm/m^2^ na síndrome de Turner; <50mm na tetralogia de Fallot) – Taquicardia ventricular	– Hipertensão arterial pulmonar – Disfunção ventricular grave (FE <30% ou NYHA classe III-IV) – Cardiomiopatia periparto com disfunção ventricular residual – Estenose mitral grave – Estenose aórtica grave sintomática – Ventrículo direito sistêmico com disfunção moderada ou grave – Dilatação aórtica grave (>45mm na síndrome de Marfan; >50mm na válvula aórtica bicúspide; ITA >25mm/m^2^ na síndrome de Turner; >50mm na tetralogia de Fallot) – Ehlers-Danlos (Re) coarctação grave – Fontan com qualquer complicação
**Taxa de eventos cardíacos maternos**	2,5% a 5%	5,7% a 10,5%	10% a 19%	19% a 27%	40% a 100%
**Risco**	Não detectado aumento do risco materno de morte e nenhum/ou leve aumento do risco na morbidade	Aumento leve do risco de morte materna ou aumento moderado da morbidade	Aumento intermediário no risco de morte materna ou aumento moderado a grave na morbidade	Aumento significativo no risco de morte materna ou grave da morbidade	Risco extremo de morte materna ou grave da morbidade

*Modificada de Regitz-Zagrosek et al.*^25^*FE: fração de ejeção; ITA: índice de tamanho da aorta; NYHA: New York Heart Association; OMS: Organização Mundial de Sáude*

As classificações III e IV da OMS contraindicam a prática dos EF na gestação, sendo que as demais classes devem ser avaliadas individualmente (Figura 2).

### 2.4. Prescrição dos Exercícios Físicos

Exercício físico é conceituado como um tipo de atividade física programada, que envolve movimento corporal repetitivo, objetivando melhorar ou manter um ou mais componentes da capacidade física.^36^

A aplicação de programas orientados para a prática do exercício, como regra geral, deve contemplar a condição fisiológica vigente na gestante, incorporando obrigatoriamente os componentes básicos: frequência, intensidade, tipo e tempo (FITT). Frequência significa a quantidade de dias por semana em que o EF deve ser praticado; tempo corresponde à duração da sessão de treinamento; o tipo pode ser aeróbico e/ou resistido; e a intensidade é a graduação do nível de trabalho, que pode ser dividida em leve, moderada e intensa.^36^

#### 2.4.1. Tipos de Exercícios e Prescrição nas Gestantes

**Exercícios aeróbicos:** São aqueles que utilizam o metabolismo de O_2_ como principal fonte de energia e demandam trabalho cardiorrespiratório para sua execução. Compreendem a movimentação em flexão e extensão de grandes grupos musculares, ocorrem de maneira rítmica e por tempo prolongado. No entanto, quando realizados em alta intensidade, podem envolver metabolismo “anaeróbico” predominante, necessitando de outros substratos energéticos para a manutenção do esforço, e com risco consequente de maior acúmulo de ácido láctico. A capacidade aeróbica, também conhecida como VO_2_, reflete a habilidade do sistema cardiovascular em transportar e entregar O_2_ para os músculos esqueléticos, além da eficiência muscular de extraí-lo da corrente sanguínea e utilizá-lo como fonte de energia para executar o exercício. ^36^

A prática regular dos exercícios aeróbicos auxilia na manutenção da capacidade funcional, no controle do peso e na prevenção de DMG e HG. Na gestação, os exercícios aeróbicos considerados seguros são os de baixo impacto, como a prática de pedalar em bicicleta estacionária e caminhada (no solo), natação e hidroginástica (aquáticos).^4^ Os EF realizados no solo, ou em meio “seco”, geralmente apresentam maior dificuldade para execução durante a evolução da gravidez, acarretam maior demanda metabólica, sobrecarga osteoarticular e dificuldade de equilíbrio, pois compreendem a sustentação do peso corporal, que aumenta progressivamente. Assim, o EF no meio aquático é considerado preferencial nesse período. Os efeitos e as recomendações para EF aquáticos serão abordados separadamente.

Como uma das principais respostas ao exercício aeróbico é o aumento da FC e da frequência respiratória, a orientação da intensidade pode ser feita com base no controle da FC, que, apesar de suas limitações nas gestantes, é o índice mais aproximado da capacidade funcional. Há vários métodos propostos para a definição da intensidade do EF aeróbico, elaborados a partir de populações saudáveis em ambos os sexos, que podem ser aplicados durante uma gestação de evolução normal, com as devidas adequações em função das sobrecargas impostas nesse período.^36^

Para gestantes saudáveis, recomenda-se a realização de EF em intensidade moderada, mas é importante ressaltar que a intensidade escolhida é de responsabilidade integrada entre o médico e o educador físico ou fisioterapeuta, podendo ser diminuída naquelas previamente sedentárias ou portadoras de comorbidades.^37^

A prescrição da intensidade pode ser estabelecida por meio do cálculo dos valores percentuais da FC máxima prevista para a idade (FCMprev) ou da FC de reserva (FCR). Valores percentuais do VO_2_ máximo e carga metabólica (MET) prevista também são opções que podem ser utilizadas (Tabela 4).^36^ Entretanto, para obtenção da FCR e do VO_2_ máximo, é necessária a realização de TE máximo, que nessa intensidade não é indicado para as gestantes. Existem outros métodos mais fáceis e práticos de orientar os EF, como, por exemplo, a classificação da percepção subjetiva do esforço de Borg^38^ (Tabela 5) e a técnica de *talk test*, em que a intensidade confortável é a que o indivíduo consegue conversar, mas não “cantar” durante os EF (Tabela 6). ^3^^,^^37^^,^^39^

**Tabela 4:** Níveis de intensidade de exercícios aeróbicos em indivíduos saudáveis

Intensidade	% VO_2_ máx.	% FCMprev	%FCR	MET* (absoluto)	Escala de Borg
Muito leve	<37	<57	<30	<2	<9
Leve	37-45	57-64	30-40	2-3,9	9-11
Moderado	46-64	65-76	40-60	4-6	12-13
Intenso	65-91	76-96	60-90	6,1-8,8	14-17
Muito intenso	>91	>96	>90	>8,9	>17

*Modificada de ACSM*.^36^

* *MET: unidade metabólica basal, que equivale ao consumo de oxigênio de 3,5 mL.Kg*^-1^.*min*^-1^ *na condição de repouso supino/sentado para a mulher não grávida. Nas sugestões apresentadas, o consumo de oxigênio (VO_2_) expresso nas unidades referidas deve ser idealmente obtido diretamente pelo teste cardiopulmonar e não estimado por equações de regressão derivadas de protocolos convencionais utilizados no teste ergométrico; % VO_2_ máx.: valores percentuais do consumo máximo de oxigênio; % FCMprev: valores percentuais da frequência cardíaca máxima prevista para a idade; % FCR: valores percentuais da frequência cardíaca de reserva; escala de Borg: escala linear de percepção do esforço, graduação de 6 a 20*.

**Tabela 5:** Escala linear de percepção subjetiva do esforço – Borg (graduada de 6 a 20)

Escala de Borg	Percepção do exercício
6	Muito fácil
7	
8	
9	Fácil
10	
11	Relativamente fácil
12	
13	Ligeiramente cansativo
14	
15	Cansativo
16	
17	Muito cansativo
18	
19	Exaustivo
20	

*Modificada de Borg*.^38^

**Tabela 6:** Métodos alternativos de prescrição de exercícios físicos aeróbicos na gestante*

Método	Descrição
Sensação subjetiva de esforço (escala de Borg)	Exercícios com autopercepção de esforço ***relativamente fácil ou ligeiramente cansativo***. Recomendado entre 12 e 13
Teste de fala (*talk test*)	Execução dos exercícios em intensidade em que a respiração seja ofegante, porém controlada, de modo que se consiga completar uma frase sem pausas
Percentuais da FCMprev	Exercícios na intensidade entre 60% e 80% da FCMprev FC alvo = percentual x FCMprev FCMprev = 208 – (0,7 × idade)** ou 220 – idade***
Frequência cardíaca de reserva (*Karvonen*)	Exercícios na intensidade entre 45% e 60% da FCR (FC pico – FC repouso) FC alvo = FC repouso + percentual × (FC pico – FC de repouso)

*FC: frequência cardíaca; FCMprev: frequência máxima prevista; FCR: frequência cardíaca de reserva*.

* *Considerar intensidade moderada*

** *Fórmula de Tanaka*

*** *Fórmula de Karvonen*.^36^

Mulheres previamente ativas devem manter os EF aeróbicos de forma habitual ou adequar para o tempo de pelo menos 30 minutos, 4 a 5 vezes por semana. As sedentárias podem iniciar, por exemplo, com 15 minutos de EF aeróbicos 3 vezes por semana e aumentar gradativamente o tempo até atingir a recomendação de 150 minutos/semana ou 30 minutos diariamente.^37^

**a) Exercícios resistidos:** representados por contrações musculares em determinados segmentos corporais, que se opõem aos movimentos ou contra uma resistência. Classificam-se em dinâmicos, quando há movimento articular associado à contração muscular, ou estáticos (isométricos), quando não há. Podem ser executados com pesos livres, em aparelhos de musculação, com faixas elásticas e até com o próprio peso corporal.^36^ Os exercícios devem ser ajustados em cada fase gestacional, adaptando-se às modificações anatômicas impostas ao corpo da mulher. EF resistidos são utilizados para aumentar tônus, força e resistência muscular, o que auxilia na adaptação às alterações posturais, redução de dores musculoesqueléticas e prevenção de eventuais quedas durante a gestação. Há demonstração que o treinamento regular de força proporciona aumento de até 14% da resistência lombar em gestantes.^40^

Alguns exemplos de exercícios resistidos são musculação, treinamento funcional, pilates e ioga.^4^ Alongamento suave e ioga também promovem relaxamento muscular, sendo particularmente úteis na melhora postural e no alívio das dores lombar e pélvica, mas devem ser realizados com cautela em virtude da maior frouxidão ligamentar característica dessa fase e consequente risco de lesões.^3^^,^^4^^,^^39^ O emprego da técnica “pilates” traz resultados satisfatórios ao preparar o abdome e o assoalho pélvico para o trabalho de parto, além de diminuir o risco de incontinência urinária. O treinamento da musculatura pélvica reduziu em até 50% a ocorrência da incontinência urinária pré-natal e 35% no período pós-natal.^41^

A intensidade do EF resistido é definida de acordo com o percentual de uma repetição máxima (RPM) que o indivíduo consegue realizar, que se reflete no número de repetições possíveis com determinada carga. A Tabela 7 descreve as faixas de intensidade para treinamento com EF resistido.^36^

**Tabela 7:** Níveis de intensidade de exercícios resistidos

Intensidade	Percentual de 1 RPM*	Número de repetições
Leve	30% a 50%	15 a 20
Moderada	50% a 70%	10 a 15
Intensa	70% a 85%	8 a 10

* *RPM: repetição máxima*.

Nas gestantes, recomenda-se a prática dos EF resistidos, três a cinco vezes por semana, com duração de 15 a 20 min, em intensidade moderada, o que corresponde à realização de duas a três séries, com 10 a 15 repetições.^3^^,^^36^^,^^37^ O número de repetições e carga deve ser ajustado de acordo com o condicionamento muscular prévio da mulher, sendo o número de repetições próximo a 10 correspondente à intensidade/carga mais elevada. Recomenda-se preferencialmente o trabalho envolvendo grandes grupos musculares, utilizando faixas elásticas ou máquinas com pesos leves em vez de pesos livres, em virtude da maior dificuldade de equilíbrio pela alteração do centro de gravidade. Cargas muito elevadas e isometria intensa não são recomendadas, pois a realização concomitante da manobra de Valsalva pode resultar em aumento da pressão intra-abdominal e diminuição consequente do fluxo sanguíneo para o feto, além de sobrecarregar mais ainda o assoalho pélvico, aumentando o risco de prolapso, incontinência anal e urinária.^19^^,^^22^

**b) Recomendações gerais de exercícios para gestantes saudáveis:** englobam a prática dos EF aeróbicos e resistidos de intensidade moderada. É importante lembrar que o tempo da sessão de treinamento deve incluir período de aquecimento e desaquecimento de intensidade leve. A prescrição de EF para a gestante conforme os componentes FITT está resumida da Tabela 8.

**Tabela 8:** Prescrição (FITT) de exercícios físicos na gestante saudável

FREQUÊNCIA	INTENSIDADE	TIPO	TEMPO
3 a 5 dias/semana	Moderada – 60% a 80% FCMprev – % de outro método validado (FCR, VO_2_) ou – 12 a 13: escala Borg ou – Correspondente a 4 – 6 MET	Aeróbico (de baixo impacto)	20 a 30 minutos por sessão (meta – acumular um total de 150 minutos/semana) 10 a 15 minutos aquecimento e desaquecimento
3 a 5 dias/semana	Moderada 2 a 3 séries e 10 a 12 repetições	Resistido	15 a 20 minutos 10 a 15 minutos aquecimento e desaquecimento

*FCMprev: frequência máxima prevista; FCR: frequência cardíaca de reserva; MET: unidade metabólica basal; VO_2:_ consumo de oxigênio*.

#### 2.4.2. Exercício Físico Aquático

A prática de exercícios em imersão tem sido documentada como benéfica e segura durante a gestação. O meio aquático proporciona flutuabilidade, que facilita a execução dos movimentos, diminuindo o peso corporal e a sobrecarga osteoarticular, com sensação de bem-estar e sem risco de quedas. Além disso, facilita a dissipação de calor, favorecendo a termorregulação, o que diminui a chance de hipertermia, cuja ocorrência é indesejada durante o EF devido à associação ao risco de malformações fetais.^42^ Dessa forma, o exercício aquático constitui a modalidade de EF de escolha nesse período, representado principalmente pela hidroginástica, que pode ser realizada incluindo componentes de treinamento aeróbico e resistido.

O principal efeito favorável da imersão na mulher grávida se deve à presença da pressão hidrostática, que é proporcional à profundidade, atua uniformemente na superfície corpórea, redistribuindo os fluidos do extravascular para o intravascular, levando à rápida expansão do volume plasmático, volume sistólico e DC, bem como aumento do fluxo uteroplacentário e da diurese. O tônus uterino e a FC fetal não apresentaram alterações durante imersão ou após os exercícios em água, demonstrando que não ocorre comprometimento da circulação uteroplacentária ou do substrato energético para o feto.^43^

Do ponto de vista metabólico, ocorrem alterações hormonais (aumento do fator natriurético e diminuição do hormônio antidiurético secundários ao estímulo de receptores) que aumentam a diurese por até 4 horas. Esse efeito contribui para a diminuição do edema periférico e menores níveis de PA após o esforço.^44^

Em comparação aos exercícios em solo (meio “seco”), observa-se que o incremento da FC, a PA e a temperatura materna são menores na água, devendo ser ajustadas às faixas de treinamento (cerca de 15 batimentos por minuto a menos). O rendimento aeróbico parece ser melhor, pois, com menor peso corporal, o consumo relativo de oxigênio é maior.^45^^,^^46^

Para tais benefícios, recomenda-se que o tórax permaneça imerso ao nível do apêndice xifoide ou logo abaixo, que a temperatura da água esteja entre 28 e 30°C (nunca exceda a 33,4°C) e a duração da atividade em torno de 45 min, especialmente para mulheres destreinadas, sugerindo-se o apoio dos pés para aquelas que não dominam a técnica natatória.

A natação também tem se mostrado segura, respeitando-se os limites de volume e intensidade recomendados para os EF na gestação. Curiosamente, um estudo recente sugere que a natação em água fria (até e acima de 20 graus, sem aquecimento artificial) pode trazer benefícios às gestantes e ao feto, pois estaria associada à diminuição do estresse e dos níveis de cortisol (aumentado ao repouso), bem como ao aumento do limiar de dor.^47^ Deve-se observar que tal estudo foi realizado com mulheres do norte europeu, mais habituadas a tal prática.

O mergulho com equipamentos (SCUBA) parece ser seguro para a mulher grávida, porém não é para o feto. No caso de descompressão brusca, a circulação pulmonar fetal não é capaz de filtrar bolhas, levando ao risco de embolia gasosa e malformações fetais. Portanto, o mergulho em profundidade deve ser evitado durante o período gestacional, estando liberada a prática tipo natação com *snorkel.*^48^

As recomendações dos componentes FITT para exercícios aquáticos durante a gestação são resumidas na Tabela 9.

**Tabela 9:** Orientações para exercícios aquáticos

FREQUÊNCIA	INTENSIDADE	TIPO	TEMPO
3 a 5 dias/semana	Moderada (para componentes aeróbicos e resistidos)	Hidroginástica ou natação	Até 45 minutos/sessão

*Temperatura da água entre 28 e 30°C; imersão até o nível ou logo abaixo do apêndice xifoide*

### 2.5. Exercícios Físicos em Populações Especiais

#### 2.5.1. Distúrbios Hipertensivos da Gravidez

Os distúrbios hipertensivos da gravidez (DHG) ocorrem em até 10% das gestações e estão associados ao aumento do risco cardiovascular ao longo da vida;^49^ além disso, apresentam fatores de risco semelhante aos da população em geral (idade materna avançada, etnia, história familiar de hipertensão e estilo de vida sedentário). A classificação dos DHG está descrita na Tabela 10.

**Tabela 10:** Classificação dos distúrbios hipertensivos da gravidez^25^

Distúrbios Hipertensivos da Gravidez	
Hipertensão preexistente (HPe)	Precede a gravidez ou se desenvolve antes da 20ᵃ semana de gestação Persiste por mais de 42 dias após o parto Pode estar associada à proteinúria
Hipertensão gestacional (HG)	Desenvolve-se após a 20ᵃ semana de gestação e se resolve por até 42 dias após o parto
Pré-eclâmpsia (PE)	Hipertensão gestacional com proteinúria significativa: >0,3 g/24 h e/ou Razão albumina-creatinina >30mg/mmol

**Tabela 11:** Índice de risco Cardiac Disease em Pregnancy (CARPREG)

Preditores de Eventos Cardiovasculares	Pontuação
Evento cardíaco prévio (falência cardíaca, ataque isquêmico transitório, infarto antes da gravidez ou arritmia)	1
Classe funcional de NYHA basal > II ou cianose	1
DOLEC (valva mitral com área <2cm^2^, valva aórtica <1,5cm^2^ ou gradiente de pico de fluxo de via de saída >30mmHg	1
Função ventricular sistólica reduzida (fração de ejeção <40%)	1

*Modificada de Martins et al*.^79^*DOLEC: doenças obstrutivas do lado esquerdo; NYHA: New York Heart Association*.

A HG afeta 5 a 8% das mulheres e é caracterizada por PA sistólica >140mmHg e/ou PA diastólica >90 mmHg, e deve ser medida em posição sentada ou em decúbito lateral esquerdo. A PE é um distúrbio multissistêmico que ocorre também de 5% a 8% das gestações, sendo mais frequente durante a primeira gestação, gravidez múltipla, mola hidatiforme, síndrome antifosfolípide, hipertensão arterial, doença renal ou diabetes preexistente. Está frequentemente associada à restrição do crescimento fetal devido à insuficiência placentária (25% dos casos), sendo causa comum de prematuridade (27%) e 4% de óbito fetal intrauterino. O principal tratamento e mais efetivo é o parto.^25^

A literatura científica demonstra que a prática regular de exercícios físicos melhora a saúde cardiovascular na gestação e pode diminuir o risco de desenvolver distúrbios hipertensivos da gravidez em até 30%.^37^^,^^49^^,^^50^ A atividade física praticada antes da gestação está relacionada a menor ocorrência de PE, com redução de 22% a 35% no risco relativo (RR) para mulheres com o nível moderado e alto de atividade física, respectivamente.^51^ Esse risco é ainda mais baixo com a atividade física combinada antes e logo no início da gestação. Ao avaliar o efeito dose-resposta da atividade física, 5 a 6 h por semana reduzem o risco de PE em até 40%, mas nenhuma redução adicional com o aumento do nível de atividade foi relatada. ^25^^,^^50^

De acordo com diretrizes brasileiras e internacionais, a prática dos EF está contraindicada em gestantes com HPe não controlada e diagnóstico suspeito ou confirmado de PE, sendo contraindicação relativa na HG.^49^^,^^50^

Não existem valores de PA estabelecidos como limites para realização de EF durante a gravidez, além de consenso entre especialistas. A Diretriz Brasileira de Reabilitação Cardiovascular preconiza que uma sessão de exercícios não deve ser iniciada quando os níveis de PA em repouso forem superiores a 160/100mmHg, e deverá ser interrompida em valores de 220/105mmHg na população cardiopata e hipertensa.^52^

Com base nesses conceitos, sugere-se que alguns cuidados merecem ser respeitados:

1. Para gestantes que estão com PA controlada (<140/90mmHg), recomendam-se EF de leve intensidade nas pacientes sedentárias, mas as fisicamente ativas poderão manter o nível moderado. Nesta condição, realizar aferições da PA (antes, durante e após o exercício), interrompendo-se as atividades quando valores acima de 160/100mmHg forem alcançados. Manobras de Valsalva devem ser evitadas rotineiramente do início até o final da gestação.
  - Primeiro trimestre: nesta fase, é comum observar-se redução dos níveis de PA, sendo que muitas mulheres tendem a não fazer uso da medicação específica. A interrupção dos medicamentos anti-hipertensivos deve ser evitada; contudo, ajustes de dosagem podem ser necessários em combinação com o aumento na ingestão hídrica. Este cuidado auxiliará na manutenção correta da hidratação e controle da temperatura corporal para realização dos exercícios. Por vezes, a redução da intensidade dos exercícios deve ser aplicada.
  - Segundo trimestre: neste período, a PA se estabiliza e volta a se elevar, podendo ser necessários novos ajustes terapêuticos. Este trimestre é o mais hemodinamicamente estável e permite discretos aumentos de carga para mulheres com peso adequado e níveis controlados de PA. Exercícios em posição supina passam a ser evitados a partir desta fase.
  - Terceiro trimestre: a compressão mecânica exercida pelo útero sobre a aorta e veia cava inferior aumenta drasticamente, havendo maior estase e edema nos membros inferiores. Esta situação favorece ao aumento da PA por retenção hídrica. A vigilância sobre os níveis de PA deve aumentar, e a intensidade dos exercícios deve diminuir. A autolimitação na intensidade do exercício, ou mesmo na adesão de sua realização, é natural neste período. Sugerimos manutenção de exercícios da musculatura pélvica, alongamentos e aeróbicos de baixa ou muito baixa intensidade, enquanto os níveis pressóricos se mantiverem dentro de limites aceitáveis (≤160/100mmHg). Se houver qualquer elevação não esperada nos níveis pressóricos, os exercícios deverão ser interrompidos.

#### 2.5.2. Diabetes Melito

O DMG apresenta complicações em curto e longo prazo para a mãe e o bebê. O diagnóstico precoce é importante para que intervenções possam ser realizadas com o objetivo de reduzir efeitos deletérios da hiperglicemia. Observa-se elevada frequência de distocias e tocotraumatismos maternos, com maior chance do desenvolvimento de HG ou PE. Em longo prazo, cerca de 50% dessas mulheres desenvolvem diabetes melito (DM) tipo 2 e os seus filhos correm risco maior de nascer pós-termo, bem como são mais propensos a desenvolver síndrome metabólica na infância e na vida adulta.^53^^–^^55^

Modificações de estilo de vida como dieta e exercícios são a base para o tratamento do DMG e importantes aliadas quando associadas ao tratamento medicamentoso. Em contrapartida, os estudos mostram que o comportamento sedentário aumenta o risco de DMG.^53^^,^^56^

Nos casos de DMG, os estudos têm demonstrado evidências de que os exercícios orientados reduzem possíveis desfechos negativos maternos e fetais. No entanto, nas gestantes com DM preexistente, tal evidência ainda não foi comprovada.^53^^,57,58^ De qualquer forma, em ambas as populações, exercícios aeróbicos de baixa a moderada intensidade e resistidos auxiliam no controle glicêmico e reduzem a necessidade de insulina, além de todos os outros benefícios físicos e psicológicos já relatados.^53^^,^^58^

Atenção especial deve ser dada às contraindicações à prática do exercício, como em casos de retinopatia pré-proliferativa, hiperglicemia sem controle, hipoglicemias sem aviso, neuropatia periférica avançada e disautonomias. Nas gestantes com DM, recomenda-se exercícios aeróbicos e resistidos, com intensidade leve a moderada, no mínimo três vezes por semana, com duração de 30 min em cada sessão.^57^^,^^59^^,^^60^

Um dos maiores riscos do exercício em pacientes diabéticas é a hipoglicemia, a qual pode ocorrer durante ou após o exercício. O monitoramento da glicemia capilar deve ser realizado antes e após os exercícios, principalmente nas gestantes que vão iniciar um programa de exercícios e após ajuste de esquema terapêutico. Para início dos exercícios, o preconizado é que a glicemia capilar esteja entre 100 e 200mg/dL. Na presença de níveis abaixo de 100mg/dL, deve-se estimular a ingestão de 15 a 30 g de carboidratos de rápida absorção e repetir a dosagem com 30 min. Não realizar exercícios físicos em jejum ou mais do que 3 h sem alimentação. Nos casos de hiperglicemia com níveis capilares acima de 250mg/dL, os exercícios estão contraindicados pelo risco de complicações como a cetoacidose diabética.^57,60^

Alguns aspectos devem ser ressaltados nas pacientes com diabetes tipo I, considerando-se o controle glicêmico quando os níveis de HbA1c forem menores que 7,5%.^61^ Não realizar EF no pico de ação da insulina e também não aplicar insulina em áreas que serão mais exigidas durante o esforço físico, devido à maior absorção no local.^57^^,^^59^^,^^60^ O DM tipo I não controlado é contraindicação absoluta para realização dos EF.

A gestação é período de desafio para a mulher que desenvolve diabetes, mas também de maior motivação para mudanças saudáveis no estilo de vida, que podem persistir após o nascimento e ajudar a prevenir o aparecimento de DM tipo 2.

#### 2.5.3. Obesidade

A gestação está incluída na lista de fatores clássicos para desencadear a obesidade, que predispõe ao DMG, DM tipo 2, HAS, DCV e ao câncer. Filhos de mães obesas também demonstram aumento na incidência de obesidade e suas implicações metabólicas e cardiovasculares. Além disso, há evidências de que mulheres obesas ou com sobrepeso têm menores taxas de início e duração da amamentação, implicando em desvantagem para o crescimento e desenvolvimento de suas crianças.^62^^–^^64^

A obesidade aumenta a probabilidade de trabalho de parto prolongado provavelmente por menor tônus miometrial, gestação pós-termo, parto cesárea, internação prolongada e infecções puerperais. Os fatores contribuintes para a maior frequência da via de parto cesárea são a desproporção cefalicopélvica e distocia por aumento de tecidos moles depositados na pelve materna.^65^ Em partos vaginais, a maior prevalência de macrossomia contribui para distocia de ombro, provocando lacerações perineais e paralisias do plexo braquial no recém-nascido.^64^

Pelo fato de a obesidade ou o ganho excessivo de peso estarem ligados ao aumento das complicações maternas e fetais durante a gestação, parto e pós parto, recomenda-se fortemente adesão à dieta equilibrada e exercício físico regular preferencialmente supervisionado.^65^^–^^67^ A principal meta durante a fase gestacional não se relaciona à perda de peso, mas, sim, ao ganho adequado durante toda a gravidez, evitando-se assim o excesso no terceiro trimestre. O peso materno é fator de risco independente para PE, que dobra a cada aumento de 5 a 7 kg/m^2^ no índice de massa corporal (IMC) pré-gestacional.^68^ O treinamento físico no período pré-natal reduz o ganho de peso e o risco de DMG para gestantes em sobrepeso e obesas.^64^

Vários estudos evidenciaram a segurança da prática de exercícios físicos em intensidade moderada ao longo da gravidez, na ausência de contraindicações médicas ou obstétricas.^3^^,^^69^ Nas gestantes portadoras de obesidade, recomendam-se exercícios aeróbicos, de leve a moderada intensidade, prescrita entre 35% e 60% da FCR, que vai depender da condição cardiorrespiratória prévia. Os benefícios do treinamento de resistência muscular também podem ser verificados neste grupo.^37^^,^^39^

Um dos aspectos de importância relaciona-se à adesão desta população à prática regular das atividades físicas, mesmo em programas supervisionados. Este desafio é o ponto-chave a ser avaliado, com intuito de evitar ou diminuir o abandono dos EF.^69^

Frente a todos os efeitos favoráveis para o binômio mãe-feto, as mulheres com sobrepeso ou obesidade devem ser encorajadas a abandonar a vida sedentária. No período do pós-parto, o incentivo para a prática de exercícios físicos e a manutenção de dieta saudável são mandatórios, não só para a perda do excesso de peso gestacional, como também para o alcance de metas de peso mais adequadas, ao serem planejadas gestações futuras.

#### 2.5.4. Atletas

O treinamento de atletas pressupõe a realização de exercícios em grande volume e alta intensidade. Além das próprias limitações impostas pela gravidez, questões éticas dificultam a realização de estudos, sem evidências robustas que demonstrem a segurança materno-fetal para a prática de exercícios em alta intensidade durante a gestação. Entretanto, algumas gestantes optam por realizar esse tipo de treinamento, inclusive mantendo competições.

Mulheres ativas que mantêm a prática de exercícios durante a gestação apresentam menor FC de repouso e maior volume sistólico, com melhor tolerância a maiores intensidades de esforço, inclusive no puerpério, facilitando o retorno às condições pré-gestacionais, eventualmente até com melhor desempenho.^70^ Tais dados são relevantes nas atletas que pretendem continuar a carreira e retomar o treinamento de alto nível em curto prazo.

Por outro lado, deve-se levar em consideração que há diminuição da capacidade residual funcional, redução do estoque de glicogênio hepático e maior dificuldade metabólica de adaptação ao exercício anaeróbico, com maior acúmulo plasmático do ácido láctico e diminuição da disponibilidade de glicose, principalmente quando o tempo de exercício é prolongado. Em comparação com mulheres sedentárias, as ativas e seus bebês apresentam peso menor, porém observa-se que isso se deve à diminuição do percentual de gordura corporal, sem prejuízo para ambos.^71^

Treinamento e provas de *endurance,* como maratonas, devem ser evitados, pois, em períodos de exercício acima de 60 min, há risco de hipertermia materna (que pode causar malformações do tubo neural se ocorrer entre a 4ᵃ e a 6ᵃ semana) e hipoglicemia fetal (identificada por diminuição transitória da reatividade do feto).^72^ Recomenda-se evitar a prática em ambientes quentes e úmidos, redobrar a atenção à hidratação e ao aporte calórico, ajustados para as necessidades metabólicas aumentadas, visando manter a homeostase para o feto.

Esportes com risco de traumatismo por colisão (basquete, futebol), por objetos (hóquei, vôlei) e por queda (saltos, equitação, ciclismo, esqui) também são desaconselhados, pois podem ocasionar descolamento de placenta e/ou hipóxia fetal devido ao traumatismo direto ou à desaceleração.^3^^,^^19^

Apesar dessas considerações, há casos conhecidos de atletas de elite que mantiveram a prática esportiva durante a gestação, inclusive competitiva, e conquistando títulos em esportes como tênis, vôlei de praia, atletismo, maratona, escalada em montanha e esqui, sem relatos de complicações materno-fetais.^65^^,^^73^^,^^74^

Entre os poucos dados da literatura, podemos citar o estudo norueguês que demonstrou que um grupo de atletas de elite que treinaram em volume e intensidade alta a partir da 17ᵃ semana apresentou benefícios para retornar à competição após o parto, sem aumento dos riscos quando comparadas com o grupo que realizou treinamento com menor carga de trabalho, sugerindo que mulheres bem treinadas poderiam manter a intensidade habitual durante a gestação.^75^ Outro estudo realizou teste de esforço até a exaustão (FC >90% da máxima prevista) em três grupos de gestantes: inativas, ativas e muito ativas (atletas), e relatou que um pequeno número de mulheres apresentou diminuição do fluxo em artéria uterina, seguido de bradicardia fetal leve e transitória (<3 min). Contudo, não se conhece o significado deste achado, tendo em vista que não houve prejuízo ao feto antes ou após, nem maior ocorrência de complicações até o parto, em comparação com as outras.^76^

De qualquer modo, os dados ainda são escassos e há necessidade iminente de mais estudos com atletas gestantes e treinamento de alta intensidade, dentro de princípios éticos e de segurança materno-fetal favorável. Apesar de alguns grupos advogarem que as recomendações para atletas poderiam ser revistas, sendo mais liberais quanto ao tipo de treinamento, ainda não há dados suficientes para a afirmação de que a prática de exercícios em alta intensidade seja segura durante a gestação. Cabe ao médico compreender o impacto psicológico e financeiro que essas condições podem causar no bem-estar e na carreira da atleta, orientando sobre os riscos de complicações e até mesmo de interrupção indesejada da gestação ou danos irreversíveis ao bebê, permanecendo a decisão compartilhada a mais acertada.

#### 2.5.5. Cardiopatias

As modificações que ocorrem durante o processo da gestação são dinâmicas, similares a uma prova de esforço, com mudanças cardiovasculares e sistêmicas que podem comprometer a estabilidade da doença cardíaca. O aumento de VS, FC, DC e fluxo sanguíneo uterino, a redução do hematócrito e a reserva cardíaca podem ser suficientes para instabilidades hemodinâmicas, não apenas no início, mas também no transcorrer de todo o período gestacional.^25^^,^^77^ As consultas ao cardiologista devem ser regulares para auxiliar no controle clínico dessas pacientes.

Embora a prática regular de exercícios físicos seja indicada para as prevenções primária e secundária das DCV, não está amplamente documentada nas gestantes cardiopatas. Os estudos são escassos com esse perfil de pacientes, visto a complexidade da maioria das DCV, tais como cardiopatias congênitas, valvopatias, hipertensão pulmonar, condições que colocam em risco a vida da mãe e do feto. A liberação dos EF nessa população deve ser baseada principalmente na avaliação do risco cardiovascular para evolução da gravidez. A literatura demonstra importantes preditores de eventos cardiovasculares maternos, tais como classe funcional pela New York Heart Association (NYHA) III e IV, lesões obstrutivas do coração esquerdo (moderadas a severas), eventos cardíacos prévios, redução da fração de ejeção do VE (<40%), regurgitação da valva atrioventricular sistêmica (moderada a severa), regurgitação da valva atrioventricular pulmonar (moderada a severa), hipertensão arterial pulmonar, uso de medicação cardíaca antes da gravidez, cianose (saturação de oxigênio <90%), tabagismo, prótese valvar mecânica, doença cardíaca cianótica corrigida ou não.^24^

Estudo interessante de Jastrow et al. com 227 mulheres cardiopatas acompanhadas por 312 gestações utilizou escore de risco denominado Cardiac Disease in Pregnancy (CARPREG) com suas variáveis descritas na Tabela 11, no seguimento e a associação com desfechos maternos e fetais. No grupo avaliado, as lesões cardíacas maternas eram predominantemente congênitas (81,4%), com escore de risco baixo ou escore = 0 em 66,3%, e risco intermediário ou escore = 1 em 33,7%. Os eventos cardíacos maternos complicaram em 7,4% das gestações, com edema pulmonar ocorrendo em maior frequência (3,8%). O escore intermediário esteve associado à maior taxa de desfechos cardíacos maternos (19,0% × 1,4%, *odds ratio* [OR] = 15,6, 95% do IC), com eventos adversos em 27,5% dos neonatos. O índice de risco CARPREG apresentou alta sensibilidade e valor preditivo negativo em relação às complicações cardíacas em gestantes com cardiopatia.^78^ Entretanto, esse índice aplicado em uma amostra composta principalmente por pacientes com cardiopatia reumática superestimou o número de eventos em gestantes classificadas como CARPREG 1 e > 1, além de subestimar o risco em pacientes gestantes de baixo risco (CARPREG 0).^79^

Existem outros índices de avaliação do risco materno-fetal, sendo considerado o mais preciso e utilizado atualmente o da OMS, descrito anteriormente. Os EF nas gestantes com risco III e IV pela OMS são considerados proscritos.^25^ Nos demais casos, sugere-se avaliação pormenorizada da cardiopatia, considerando também a condição cardiorrespiratória da gestante e as modificações fisiológicas peculiares de cada período gestacional, para análise do risco e benefício da prática de EF para a mãe e o feto. A interação entre a cardiologia e a obstetrícia é muito importante na avaliação e liberação dessas pacientes para realização do EF.

As provas funcionais como TE e TCPE têm indicação controversa nesse cenário, com algumas sugestões para provas submáximas e com utilização em situações específicas (doença valvar e cardiopatias congênitas).^25^

Na orientação dos EF, os níveis de FC, PA e a saturação periférica de oxigênio pela oximetria de pulso podem nortear o tipo e a intensidade do exercício aplicado por profissionais especializados e treinados. É fundamental dar atenção especial aos sinais e sintomas de alerta para interrupção das atividades: cansaço, dispneia, sinais de baixo débito, dentre outros já descritos.

As gestantes com DCV que apresentam potencial para complicações (descritas na Tabela 12) devem ser preferencialmente acompanhadas por equipe multidisciplinar, englobando medicina materno-fetal, cardiologia, cirurgia cardiovascular, anestesiologia, neonatologia e encaminhadas a um centro de atendimento terciário.^80^

**Tabela 12:** Principais complicações de gestantes com doença cardiovascular

Condição Cardiovascular	Complicações Potenciais
Hipertensão arterial	– Pré-eclâmpsia; síndrome Hellp*
Estenose mitral	– Taquiarritmias com baixo débito, edema pulmonar
Insuficiência mitral	– Arritmia, edema pulmonar
Estenose aórtica	-Baixo débito com queda da pré-carga
Insuficiência aórtica	– Geralmente bem tolerada – Insuficiência cardíaca congestiva
Estenose pulmonar Insuficiência pulmonar	– Geralmente bem tolerada, – Edema pulmonar se insuficiência cardíaca direita
Estenose tricúspide	– Bem tolerada
Insuficiência tricúspide	– Bem tolerada
Cardiomiopatia	– Arritmia; insuficiência cardíaca congestiva; edema pulmonar
Aneurisma de aorta	– Dissecção de aorta, rotura

*Modificada de Adam et al*.^80^

* *Hellp: hemólise, elevação de enzimas hepáticas e plaquetopenia*.

No Brasil, a doença reumática é a cardiopatia mais frequente em gestantes, acometendo principalmente as valvas mitral e aórtica.^24^ As cardiomiopatias apresentam baixa prevalência nessa população, e sua apresentação heterogênea faz com que as orientações sejam individualizadas. ^24^^,^^25^^,^^52^

A orientação da atividade física nas doenças valvares dependerá da valva acometida e do grau de comprometimento. Estenoses valvares, particularmente do lado esquerdo, apresentam maior risco de complicações maternas do que as regurgitações valvares, em que DC, vasodilatação e taquicardia provocados pela gestação não são bem tolerados.^81^ Deve-se analisar também tamanho das cavidades, função ventricular, PA pulmonar, associação com outras cardiopatias, fibrilação atrial, insuficiência cardíaca, tromboembolismo e endocardite prévios, que são considerados fatores de prognóstico. Seguindo a classificação de risco da valvopatia para a gravidez (Tabela 13), orientada pelo Departamento de Cardiologia da Mulher em seu Posicionamento,^24^ recomenda-se que pacientes com risco intermediário e alto não devem ser liberadas para realização dos EF em virtude da gravidade da cardiopatia e do risco consequente para mãe e o feto.

**Tabela 13:** Classificação do risco das valvopatias para a gravidez

Risco Alto	Risco Intermediário	Risco Aceitável
Estenose mitral grave	PB com disfunção moderada	Valvopatia discreta
Estenose aórtica grave	Estenose pulmonar grave	PB sem disfunção
PB estenótica/calcificada	PM	Valvopatia + FEVE normal
PM com disfunção	PM mitral > risco PM aorta	Valvopatia sem fatores desfavoráveis
Valvopatia + PAP ≥50mmHg	Insuficiência aórtica + doenças da aorta	
Insuficiência aórtica + doenças da aorta	Síndrome de Marfan (DAorta entre 40 e 45mm)	
Síndrome de Marfan (DAorta >45mm)	Valva aórtica bicúspide (DAorta entre 45 e 50mm)	
Valva aórtica bicúspide (DAorta >50mm)	Necessidade de anticoagulantes	
Valvopatia + FEVE <35%		

*Modificada de Avila et al*.^24^*DAorta: diâmetro da aorta ascendente; FEVE: fração de ejeção do ventrículo esquerdo; PAP: pressão da artéria pulmonar; PB: prótese biológica; PM: prótese metálica. Considera-se estenose aórtica ou mitral grave: área valvar ≤1cm*^2^.

Nas lesões valvares de grau discreto, na ausência de fatores desfavoráveis e capacidade funcional dentro do previsto para a idade, sugere-se a realização dos EF de leve intensidade. É aceitável a manutenção dos EF de moderada intensidade, apenas nas gestantes com regurgitações valvares leves sem repercussão hemodinâmica, como nas insuficiências mitral e aórtica, principalmente nas pacientes que já realizavam EF anteriormente.

Ressalta-se a importância do acompanhamento dos sintomas, achados ecocardiográficos e peculiaridades de cada período gestacional. No terceiro trimestre, com as próprias mudanças cardiovasculares e respiratórias fisiológicas da gestação, além do aumento do peso, os EF devem ser reavaliados pelo risco de parto prematuro e retardo do crescimento intrauterino. É importante sempre lembrar de que o exercício físico é importante para a mãe, mas pode eventualmente ser prejudicial ao feto.

### 2.6. Cuidados na Realização dos Exercícios Físicos na Gestação e Critérios de Interrupção

A adoção de medidas gerais para a manutenção de estilo de vida saudável é fundamental na gestação, como manter nutrição e repouso adequados, realizar atividade física e evitar o consumo de álcool, o uso de medicamentos desnecessários e o tabagismo ativo ou passivo.

A gestante necessita ter o conhecimento de alguns cuidados e peculiaridades na realização dos EF, objetivando evitar complicações que são mais evidentes em determinados períodos da gestação. A atenção com a dieta é importante, o gasto calórico do exercício deve ser estimado e balanceado com a ingestão adequada de calorias, ressaltando-se a necessidade de atenção aos sinais e sintomas de hipoglicemia. Evitar exercícios em ambientes quentes e úmidos, especialmente durante o primeiro trimestre. Manter hidratação adequada, com ingestão de líquidos isotônicos antes e após os exercícios.

Deve-se ter atenção especial com os movimentos de alongamento e mudanças bruscas na realização dos exercícios, a partir da 10ᵃ semana, quando aumentam os níveis de relaxina e o risco de lesões.^19^ Os exercícios na posição supina podem resultar em retorno venoso diminuído e hipotensão em 10% a 20% de todas as grávidas, causando lipotimia ou síncope, principalmente após a 20ᵃ semana. Não existem evidências suficientes para contraindicar ou comprovar segurança nesse tipo de exercício.^82^ Caso ocorram esses sintomas, realizar os exercícios em decúbito lateral ou em pé. Evitar também os exercícios abdominais pelo risco de aumento da diástase abdominal.

Não existe período determinado para a interrupção da prática dos EF na gestação. Torna-se recomendável estimular os EF de leve intensidade ao final do terceiro trimestre e orientar quanto aos sinais de trabalho de parto^37^ uma vez que há naturalmente diminuição da atividade física nessa fase. Recomenda-se interrupção dos EF e consulta ao médico especialista caso apareçam sintomas de desconforto, tais como respiração curta e mantida que não resolve com repouso; cefaleia importante; contrações uterinas regulares e dolorosas; sangramento vaginal; perda contínua de fluido amniótico; desmaio ou tontura persistente; dor torácica; palpitações; distúrbios visuais; náuseas e vômitos persistentes; fraqueza muscular afetando o equilíbrio, entre outros.^3^

### 2.7. Adaptações e Recomendações para a Prática de Exercícios Durante a Pandemia do Novo Coronavírus

O Ministério da Saúde incluiu gestantes e puérperas no grupo de maior risco para evolução desfavorável à COVID-19. Não há dados de que a gravidez aumente a suscetibilidade à infecção pelo novo coronavírus (SARS-CoV-2, *Severe Acute Respiratory Syndrome*).^83^ Em revisão sistemática, incluindo 18 artigos, no período de fevereiro a março de 2020, em 108 gestantes infectadas com o SARS-CoV-2, febre esteve presente em 68% dos casos, tosse em 34% e dispneia em 12%.^84^

As adaptações fisiológicas da gravidez podem agravar alguns sintomas decorrentes da COVID-19. Deslocamento do diafragma, diminuição da capacidade pulmonar total e da complacência torácica contribuem para evolução pior em pacientes com pneumonia por COVID-19, acarretando hipóxia e comprometimento fetal. Além das alterações respiratórias da gestação, é importante destacar as modificações cardiovasculares e hematológicas, como a elevação da FC, menor resistência vascular sistêmica e estado de hipercoagulabilidade. Esses fatores têm implicações maiores na gestante cardiopata que adquire a COVID-19.^85^

Dessa forma, a prática de EF em gestantes na pandemia requer cuidados adicionais. Recomenda-se a adoção de rotina de exercícios a serem realizados preferencialmente em domicílio para redução do risco de contaminação. A intensidade de leve a moderada é geralmente segura na gestante saudável e, como regra geral, certamente contribui para modular o estresse emocional, além de todos os benefícios já relatados. As orientações gerais para as gestantes na realização dos EF em âmbito do domicílio consistem em lugar com espaço adequado, arejado e com temperatura agradável, uso de roupas leves e tênis, hidratação e alimentação adequadas. A realização de alguns exercícios pode ser adaptada com o uso de materiais domésticos do dia a dia.

Para as gestantes que já praticavam atividade física regular, torna-se mais fácil mantê-la na pandemia, pois já adquiriram conhecimento necessário quanto à execução dos EF, à percepção de esforço e aos sintomas de alerta para interrupção. Para as sedentárias, é importante que a orientação da importância dos EF ocorra desde a primeira consulta e que sejam prescritos inicialmente exercícios de leve intensidade.

A manutenção da atividade física domiciliar, principalmente para a gestante portadora de comorbidades ou sedentária, deve ser se possível supervisionada por profissional de educação física ou fisioterapia, em teleatendimento caracterizado nas formas de teleconsulta, teleaula e telemonitoramento nesse momento de pandemia, conforme autorizado pelos respectivos conselhos de especialidade. Caso o profissional julgue necessário e com base sempre no benefício e segurança das gestantes, a primeira avaliação poderá ser presencial, seguindo as medidas preventivas e de assepsia.^86^^,^^87^

Considerando-se o relaxamento da quarentena de forma heterogênea no território nacional, houve a liberação de alguns espaços públicos – a gestante deve dar preferência a lugares abertos e com menor circulação de pessoas, implicando consequentemente menor risco de contaminação. Existe consenso quanto ao uso de máscaras faciais fora do domicílio adicionalmente à manutenção do distanciamento social, especialmente para a caminhada em ruas ou condomínios. Estudo holandês recente sugeriu distância de 4 a 5m a ser obedecida em pessoas que caminham uma atrás da outra, distância de 10m para pessoas que correm ou andam de bicicleta lentamente e distância de 20m ao andar de bicicleta rapidamente.^88^

Em academias de edifícios, deve se adotar a conduta de liberação regional, com a recomendação de reserva exclusiva de horário para os moradores da mesma unidade habitacional, com higienização habitual de aparelhos com álcool 70% em ambiente arejado. Atualmente, em muitas cidades, as academias estão abertas com horários limitados e respeitando os protocolos de segurança recomendados.

Caso a gestante seja acometida pela COVID-19, a orientação de qualquer retorno à atividade física regular deverá acontecer após reavaliação médica e realização de ECG, tendo em vista a possibilidade de lesão miocárdica e arritmias secundárias ao quadro viral.^89^ Naquelas que apresentaram evolução clínica mais grave, recomenda-se consulta com o obstetra, para minuciosa avaliação materno-fetal, e com cardiologista, para avaliação de complicações cardiovasculares e necessidade de outros exames complementares, como ecocardiograma transtorácico, eletrocardiografia dinâmica (*holter*), dentre outros.

## 3. Exercícios Físicos no Pós-Parto

A realização de EF durante a gravidez aumenta a probabilidade da manutenção no pós-parto, com benefícios na qualidade de vida em curto e longo prazo. As mulheres habitualmente reduzem a prática de EF após o parto, em virtude das dificuldades enfrentadas nesse período com o nascimento do bebê. O sedentarismo contribui para o sobrepeso e a obesidade, com repercussão no aparecimento de outras comorbidades, como DM e DCV.^90^ Aproximadamente 25% das mulheres permanecem com aumento do peso, que é considerado como ganho >4kg em 1 ano após o parto.^91^ Os EF no período pós-parto são importantes para redução do peso; no entanto, terapias adjuvantes devem ser consideradas. Algumas revisões sistemáticas mostraram efeito maior sobre a perda de peso em estudos que incluíram componente dietético. Intervenções dietéticas mais intensivas e programas de atividades mais estruturados que incorporam monitores de FC foram associados à maior perda de peso.^92^^–^^94^

A importância da realização dos EF nessa fase deve ser enfatizada pelo obstetra, pontuando os benefícios na condição cardiorrespiratória, a redução da fadiga, a melhora na qualidade do sono e a redução do risco de depressão.^95^^–^^96^ Ressalta-se que a depressão pós-parto é condição prevalente que afeta aproximadamente 10% a 15% das mulheres no primeiro ano de nascimento.^97^ As mulheres relatam maior sensação de bem-estar e melhora da qualidade de vida com a prática de EF.^98^^,^^99^

A rotina de exercícios no pós-parto deve retornar gradualmente, em uma fase que a prática seja segura, a depender do tipo de parto (vaginal ou cesariano) e se houve complicações cirúrgicas. Orienta-se o retorno aos EF em torno de 6 semanas após o parto cesariano e em torno de 4 semanas após o parto vaginal. Aconselha-se que a progressão seja mais lenta se houver desconforto ou na presença de outros fatores, como anemia e infecção de ferida operatória. As pacientes que já praticavam EF devem reduzir a intensidade nos primeiros meses e evoluir gradualmente.^95^^,^^99^

A prática de exercícios aeróbicos de leve a moderada intensidade pode ser realizada em mulheres que estão amamentando, sem prejuízo na produção do leite e no crescimento da criança. O ideal é realizar os EF após a amamentação, para evitar o desconforto provocado pelo ingurgitamento das mamas e da mesma forma com proteção para a adequada sustentação. Mulheres que estão nessa fase devem manter hidratação abundante, observando inclusive a presença de volume satisfatório de diurese clara, além de garantir que a ingestão calórica não esteja abaixo de 25% das calorias consumidas e manter perda de peso de até 450g por semana. Estima-se o gasto calórico da amamentação em aproximadamente 600kcal/dia.^100^^,^^101^ Os EF de alta intensidade podem estar associados ao aumento de ácido láctico no leite materno e alterar o paladar do leite. Caso a mãe perceba que o bebê está rejeitando o leite materno, deve reduzir o nível do EF.^102^

Os exercícios resistidos estão liberados, mas são necessários cuidados para os que envolvem flexão do tronco (abdominais tradicionais), pelo risco do aumento da diástase dos músculos retos abdominais. Pilates é opção adequada para o treinamento da musculatura profunda do abdome e do assoalho pélvico, contribuindo para o corpo da mulher retornar ao seu estado pré-gestacional. O comprometimento da musculatura pélvica pode contribuir para o aparecimento de incontinência urinária e fecal, além de disfunção sexual. É importante realizar o treinamento da musculatura pélvica desde a gestação. Os exercícios de pular devem ser evitados no pós-parto pela fragilidade do assoalho pélvico.^103^

Os EF promovem benefícios na gestação, no puerpério e a longo prazo. As mulheres que mantêm a prática apresentam menor chance de permanecer com o excesso de peso adquirido na gravidez, desenvolver depressão e complicações metabólicas e cardiovasculares.^104^ Todos os profissionais de saúde responsáveis pelo acompanhamento das gestantes devem estimular e orientar a prática do EF com segurança.

### 3.1. Particularidades no Pós-Parto da Paciente com Cardiopatia

Algumas particularidades de importância acontecem no pós-parto, especialmente para as gestantes cardiopatas, pois, na fase imediata, ocorre alteração significativa da volemia, que pode levar à descompensação clínica. Após o período expulsivo, ocorre aumento súbito do retorno venoso, que se deve à autotransfusão do plexo uterino, à descompressão do fluxo da veia cava inferior e à redução da capacidade do sistema venoso. Além disso, a resistência vascular periférica está aumentada pela contração sustentada do útero, ocluindo os vasos que se abrem na superfície materna da placenta. A autotransfusão contínua que ocorre durante 24 a 72 h após o parto representa alto risco de congestão pulmonar na mulher cardiopata.^105^

De modo geral, os padrões de alteração do volume sanguíneo materno durante o trabalho de parto, o período expulsivo e o puerpério obedecem às seguintes fases: (1) hemoconcentração durante o trabalho de parto, que é variável com o grau de atividade uterina e de desidratação materna; (2) redução do volume sanguíneo, que ocorre durante e imediatamente após o parto proporcionalmente à quantidade de sangue perdida; (3) elevação imediata e transitória do volume sanguíneo, ocorrendo após a dequitação placentária e atribuída ao influxo de líquido para o espaço intravascular, devido ao esvaziamento uterino; (4) discreta elevação do volume sanguíneo entre o segundo e o terceiro dia do pós-parto, secundária ao aumento transitório da secreção de aldosterona; (5) redução do volume plasmático após uma semana do parto, de modo que o volume sistólico materno pode apresentar uma discreta queda nesse período, normalizando-se em curto prazo.^105^

Como sabemos, exercício e dieta saudável pós-parto promovem a perda de peso, o que pode melhorar ou prevenir riscos futuros relacionados à obesidade, como DM e HAS.^64^^,^^106^ No entanto, não há dados robustos na literatura sobre EF no pós-parto de pacientes com cardiopatia ou com complicações durante a gestação. Sendo assim, a prescrição dos EF segue as diretrizes e os princípios prescritos para as cardiopatias específicas, adaptados para minimizar os riscos, lesões maternas e maximizar os benefícios.^24,52^

As complicações cardíacas mais frequentes no intraparto e puerpério de parturiente cardiopata são: PE, insuficiência cardíaca, edema agudo pulmonar, arritmias, tromboembolismo e dissecção de aorta. Entre as obstétricas, incluem-se principalmente hemorragia e infecções.^24^ No caso de parto vaginal, deve-se avaliar o grau de traumatismo perineal. Após parto cesariano, outros desfechos devem ser analisados, tais como distúrbios cardiorrespiratórios, tromboembolismo, dor e necessidade de analgesia, cicatrização anormal de feridas, neuropatia e incontinência.^107^ Os EF não devem ser prescritos no pós-parto até controle da doença cardiológica ou das complicações clínicas e cirúrgicas e avaliação do risco-benefício da realização dos EF, seguindo o intervalo mínimo de 4 semanas após o parto vaginal e de 4 a 6 semanas após o cesariano. A deambulação precoce e o retorno às atividades físicas normais da pré-gravidez, assim que clinicamente seguros, podem reduzir as comorbidades associadas ao estilo de vida sedentário pós-cirúrgico.^108^^–^^110^

As atividades físicas deverão ser retomadas em concordância com o obstetra. Na presença de doenças e ou complicações cardiovasculares, a avaliação conjunta do cardiologista torna-se importante.^111^^,^^112^ Em virtude da escassez de dados sobre exercício físico no pós-parto de pacientes com cardiopatia, a maioria das orientações consiste em extrapolações das diretrizes de reabilitação cardiovascular. Sendo assim, mais estudos são necessários para o atendimento individualizado e preciso a essa população específica.
