# Supplementary material for: Posicionamento sobre Exercícios Físicos na Gestação e no Pós-Parto – 2021
Source: Arq Bras Cardiol. 2021 Jul 15;117(1):160–80. [Article in Portuguese] doi: 10.36660/abc.20210408 (PMC8294738; doi:10.36660/abc.20210408)
Supplement: Translation

# Translation: Position Statement on Exercise During Pregnancy and the Post-Partum Period – 2021

**Authors:** Milena dos Santos Barros Campos, Susimeire Buglia, Cléa Simone Sabino de Souza Colombo, Rica Dodo Delmar Buchler, Adriana Soares Xavier de Brito, Carolina Christianini Mizzaci, Roberta Helena Fernandes Feitosa, Danielle Batista Leite, Carlos Alberto Cordeiro Hossri, Lorena Christine Araújo de Albuquerque, Odilon Gariglio Alvarenga de Freitas, Gabriel Blacher Grossman, Luiz Eduardo Mastrocola

**Affiliations:** 1 AracajuSE Brazil Hospital São Lucas, Rede D’Or, Aracaju, SE – Brazil; 2 AracajuSE Brazil Hospital Universitário de Sergipe, Aracaju, SE – Brazil; 3 São PauloSP Brazil Instituto Dante Pazzanese de Cardiologia, São Paulo, SP – Brazil; 4 São PauloSP Brazil Hospital do Coração (HCOR), São Paulo, SP – Brazil; 5 CampinasSP Brazil Faculdade de Medicina São Leopoldo Mandic, Campinas, SP – Brazil; 6 São PauloSP Brazil Ergometria DASA, São Paulo, SP – Brazil; 7 Rio de JaneiroRJ Brazil Instituto Nacional de Cardiologia, Rio de Janeiro, RJ – Brazil; 8 Rio de JaneiroRJ Brazil Hospital Copa Star – RDSL, Rio de Janeiro, RJ – Brazil; 9 GoiâniaGO Brazil Universidade Rio Verde (UniRV), Goiânia, GO – Brazil; 10 GoiâniaGO Brazil Secretaria Municipal de Saúde, Goiânia, GO – Brazil; 11 RecifePE Brazil Real Hospital Português, Recife, PE – Brazil; 12 RecifePE Brazil Pronto Socorro Cardiológico de Pernambuco (PROCAPE), Recife, PE – Brazil; 13 Belo HorizonteMG Brazil Minascor Centro Médico, Belo Horizonte, MG – Brazil; 14 Porto AlegreRS Brazil Hospital Moinhos de Vento, Porto Alegre, RS – Brazil; 15 Porto AlegreRS Brazil Clínica Cardionuclear, Porto Alegre, RS – Brazil

**Table:** Position Statement on Exercise During Pregnancy and the Post-Partum Period – 2021

The report below lists declarations of interest as reported to the SBC by the experts during the period of the development of these statement, 2020.	
Expert	Type of relationship with industry
Adriana Soares Xavier de Brito	Nothing to be declared
Carlos Alberto Cordeiro Hossri	Nothing to be declared
Carolina Christianini Mizzaci	Nothing to be declared
Cléa Simone Sabino de Souza Colombo	Nothing to be declared
Danielle Batista Leite	Other relationships Any economically relevant equity interest in companies in the healthcare or education industry or in any companies competing with or supplying to SBC: – Ergometry Course: GEFE
Gabriel Blacher Grossman	Nothing to be declared
Lorena Christine Araújo de Albuquerque	Nothing to be declared
Luiz Eduardo Mastrocola	Nothing to be declared
Milena dos Santos Barros Campos	Nothing to be declared
Odilon Gariglio Alvarenga de Freitas	Nothing to be declared
Rica Dodo Delmar Buchler	Nothing to be declared
Roberta Helena Fernandes Feitosa	Nothing to be declared
Susimeire Buglia	Nothing to be declared

## 1. Introduction

The Department of Ergometry, Exercise, Nuclear Cardiology and Cardiovascular Rehabilitation (DERC), through its commission on Health and Diagnosis of Cardiovascular Diseases in Women and in association with the Sports Cardiology Study Group (GECESP) and the Cardiopulmonary Rehabilitation and Metabolic Study Group (GERCPM), has prepared this position paper in accordance with Brazilian Society of Cardiology norms to guide health professionals regarding the prescription of physical exercise during pregnancy and the postpartum period, as well as to strengthen the relationship between specialists in related areas (especially clinicians, cardiologists and obstetricians), including exercise management in pregnant athletes and pregnant women with comorbidities. At the end of this document, details about exercising during the current severe acute respiratory syndrome coronavirus 2 (SARS-CoV-2)/coronavirus disease-2019 (COVID-19) pandemic will be addressed.

Exercise has proven benefits for maternal and fetal health, although women, especially those with comorbidities, often curtail physical activity or remain sedentary during pregnancy due to fear of clinical and obstetric complications. Thus, it should be made clear that exercise has a number of advantages for pregnant women, such as improved functional capacity, reduced fatigue, lower risk of depression, excessive weight gain prevention, and better control of metabolic and cardiovascular disorders (eg, preeclampsia, hypertensive disorders and gestational diabetes mellitus). Preeclampsia, hypertensive disorders and gestational diabetes mellitus are now considered emerging or sex-specific risk factors and are associated with an increased risk of cardiovascular disease (CVD).

Planned consultations with an obstetrician represent a great opportunity to raise awareness about and encourage a healthier lifestyle. In general, women who are active during pregnancy continue to be active in the postpartum period, enjoying the benefits of exercise on long-term cardiovascular health. During the COVID-19 pandemic, sedentary lifestyles have increased, and exercise should be recommended more intensely for pregnant women, including the usual precautions to prevent contamination and the spread of the virus. Pregnant women, especially those with comorbidities, should be referred to specialists to help control risk factors for CVD and receive exercise prescriptions.

This paper will describe the benefits and indications of exercise during pregnancy and the postpartum period, the standardization of clinical assessment prior to exercise prescription, guidance about prescription, criteria for stopping exercise and special conditions during COVID-19 pandemic.

## 2. Exercise During Pregnancy

### 2.1. Benefits and Indications

Pregnancy causes hormonal and anatomical changes that lead to lumbar hyperlordosis, loosening of the pelvic ligaments, fluid retention in connective tissue and increased body weight. Consequently, overload in the lumbar spine and low back pain occur in approximately 60% of pregnant women, negatively interfering in sleep quality, physical disposition, work performance, social life, domestic activities, and leisure.^1^ There is also a tendency to curtail physical activity during pregnancy, especially in the third trimester, which worsens the anatomical implications of pregnancy. Obstetrics/gynecology and cardiology groups have published recommendations and guidelines about exercise during pregnancy to promote maternal, fetal and neonatal health.^2^^–^^4^ Exercise reduces fat mass percentage, increases the transfer of oxygen (O2) and reduces diffusion of carbon dioxide (CO2) through the placenta, favoring fetal development. Additionally, regular exercise decreases the risk of gestational diabetes mellitus (GDM) by approximately 50%, and the risk of preeclampsia, gestational hypertension, excessive weight gain and depression by up to 40%.^5^^–^^8^

Pregnant women with no disorders should begin exercising as soon as they feel ready, considering that fatigue and the unwanted symptoms of early pregnancy can be disruptive. Supervised exercise is considered safe for the mother and the fetus, with no reported increase in congenital anomalies, premature births or low birth weight.^9^^–^^12^ It is important for the patient to be evaluated by an obstetrician and/or cardiologist before begnning an exercise program.

Table 1 shows the recommendations for exercise during pregnancy from a recently published international guideline, describing their evidence levels and quality.^3^ The strength of the recommendations was determined using the Grading of Recommendations Assessment, Development and Evaluation system^13^, classifying them as strong or weak according to: (1) balance between benefits and hazards, (2) overall evidence quality, (3) outcome importance, (4) resource usage (cost), (5) viability, and (6) acceptability.

**Table 1:** Recommendations for exercise during pregnancy, including the quality and strength of the evidence

Indications	Recommendation	Evidence quality
All women without contraindications should be physically active throughout pregnancy (subgroups a, b, c)	Strong	Moderate
Previously inactive women	Strong	Moderate
Women with gestational diabetes	Weak	Low
Pre-gestational BMI ≥ 25 kg/m^2^	Strong	Low
Pregnant women should have a minimum of 150 min of moderate-intensity exercise/week, totaling at least 3 days/week	Strong	Moderate
The exercise regimen of pregnant women should include aerobics and resistance exercise of varying intensities, to which stretching and yoga can be added	Strong	High
Daily pelvic floor training (Kegel exercises) to reduce the risk of urinary incontinence	Weak	Low

*Adapted from Mottola et al.**^3^**BMI: body mass index*

### 2.2. Cardiovascular and Respiratory Adaptations at Rest and during Exercise

Knowledge of cardiovascular and respiratory adaptations is of great importance for understanding common cardiorespiratory symptoms during pregnancy and for assisting in the differential diagnosis of symptoms related to decompensation of previously stable heart diseases, as well as for managing exercise during this period.

**Rest:** In pregnancy, blood volume increases secondary to an increase in plasma and red blood cells, leading to hemodilution, especially toward the end of the gestational period. Even at rest, there is an increase in stroke volume and heart rate (HR), causing an increase in cardiac output.^14^^,^^15^ There is also a change in cardiac excitability, leading to a greater occurrence of extrasystole. Increased estrogen promotes a decrease in vascular tone, including a decrease in peripheral vascular resistance and a consequent reduction in blood pressure (BP), especially diastolic BP. Although peripheral vascular resistance remains low, fluid variations near delivery cause BP lability and promote lower limb edema during this stage.^16^ These changes, which usually produce a greater sensation of palpitation at rest and post-exercise dizziness, should be considered predisposing factors for the decompensation of pre-existing heart diseases.^17^

Increasing oxygen levels are an important mechanism of maternal adaptation, since they facilitate transfer to the fetus through the placenta. The increased metabolic demands of the fetus, uterus and maternal organism result in increased oxygen consumption (VO2), carbon dioxide production and basal metabolic rate, causing hyperventilation, which is also influenced by progesterone. There is a significant increase in minute ventilation (V_E_) and tidal volume, with an increase in the partial pressure of O2 in arterial blood and a decrease in the partial pressure of CO2. Residual functional capacity decreases, mainly due to displacement of the diaphragm, which increases the sensation of respiratory discomfort, even at rest. These changes explain the “physiological dyspnea” observed in about 60% to 70% of healthy pregnant women, common around the 30th week, as well as a reduced ability to hold their breath, which limits activities such as diving and anaerobic exercise (eg, sprinting).^18^ Physiological adaptations during pregnancy are described in Figure 1.

**Figure 1:** Physiological changes during pregnancy. CO: cardiac output; HR: heart rate; BP: blood pressure; PVR: peripheral vascular resistance; VE: minute ventilation.

**Exercise:** During exercise, systolic volume, cardiac output, HR and V_E_ also increase, and an increase in aerobic capacity and higher performance can already occur during the first trimester of pregnancy. Although the HR of pregnant women is higher at rest, it does not increase the same way during exercise, and VO2 does not increase proportionally with higher loads, especially when the effort includes supporting body weight. Thus, functional capacity is limited as pregnancy progresses. The increase in V_E_ exceeds the increase in VO2, but the arteriovenous difference in O2 decreases, which provides greater delivery of oxygen to the fetus. However, during high-intensity exercise the blood flow may be diverted from the uterus to the muscles, which can be harmful to fetal development.^18^^,^^19^

From an anatomical point of view, there is an increase in the left ventricular cavity, although wall thickness is not increased. A study of 105 pregnant women found that 35% developed an increase in left ventricular trabeculation, with 8% fulfilling the criteria for non-compacted myocardium. Similar changes have been reported in other situations where there is an increase in cardiac output, such as in athletes (which is reversible after returning to baseline hemodynamic conditions), and should not be confused with pathological changes.^20^

### 2.3. Exercise Assessment

Recommendations for and impediments to exercise (Figure 2) during pregnancy are usually determined by an obstetrician after clinical evaluation and identification of contraindications, such as pre-existing diseases, medical or obstetric complications, etc. (Table 2).^3^ Women with CVD should also be accompanied by a cardiologist. Currently, the partnership between cardiology and obstetrics/gynecology allows the early identification and modification of risk factors for CVD.^21^

**Figure 2:** Exercise assessment for pregnant women. ECG: electrocardiogram; WHO: World Health Organization. Clinical Assessment (Anamnesis and Exercise)

**Table 2:** Contraindications for physical activity during pregnancy

OBSTETRIC CAUSES	
ABSOLUTE	RELATIVE
Membrane rupture	History of premature birth
Premature labor	Recurrent loss of pregnancy
Placenta previa after 28 weeks of gestation	Twin pregnancy >28 weeks
Multiple pregnancy (triplets or higher-order)	
Unexplained persistent vaginal bleeding	
Cervical weakness	
Intrauterine growth restriction	

CLINICAL CAUSES	
ABSOLUTE	RELATIVE
Chronic arterial hypertension (uncontrolled)/ preeclampsia	Gestational hypertension
Severe cardiovascular disease (WHO III-IV)	Mild/moderate cardiovascular disease (WHO I-II)
Restrictive lung disease	Mild/moderate lung disease
Type I diabetes mellitus, thyroid disease (uncontrolled)	Extreme obesity, malnutrition or eating disorder Symptomatic anemia

*WHO: World Health Organization.*

The Physical Activity Readiness Medical Examination, developed by the Canadian Society for Exercise Physiology for exercise evaluation and guidance during pregnancy, has been very well accepted by medical societies worldwide for its practicality. It includes the following sections: **a)** a pre-exercise health survey (completed by the patient); **b)** exercise contraindications; **c)** a health assessment (completed by the attending physician) to be used by the exercise physiologist; **d)** instructions for prescribing aerobic and muscle conditioning exercises, thus providing recommendations for a safe and personalized prescription, as well as indicators for exercise cessation.^22^

After a thorough anamnesis and clinical examination, exercise can be recommended and supervised during pregnancy. Routine cardiac exams are not indicated for all pregnant women, only those whose clinical condition warrants them. Although electrocardiography (ECG) is not part of the prenatal routine, it should be used when investigating heart disease, monitoring pregnant women with previous CVD and assessing arrhythmias.^23^ ECGs are also recommended for patients who have clinical and obstetric contraindications to exercise (Table 2).

Transthoracic ECG is the preferred imaging method when CVD is suspected; it is non-invasive, easy to perform, and widely available.^24^ It is recommended for patients with previous heart disease, symptoms suggestive of heart disease, or abnormal previous ECG findings.

The one-repetition maximum (1RM) strength test is not recommended for functional assessment in pregnant women. If absolutely necessary for cardiovascular evaluation and after excluding contraindications, the patient can perform a submaximal exercise test (up to 85% of the maximum expected HR), which is considered safe by the Brazilian and European Societies of Cardiology. However, due to a lack of studies it cannot be recommended for characterizing ischemic heart disease. Performing an ECG during a submaximal exercise test on a bicycle ergometer can improve specificity in the detection of coronary artery disease, (ie, by adding imaging to the findings of the submaximal exercise test). Pharmacological stress with dobutamine during pregnancy is contraindicated.^24^^,^^25^

Regarding exercise prescription, especially for patients at higher cardiovascular risk, a submaximal exercise test is more efficient when associated with expired gases analysis, ie, the cardiopulmonary exercise test (CPET), which determines functional limitations and the ventilatory threshold, providing a more accurate, safe and objective prescription.^26^^,^^27^ To evaluate pregnant women, there is no good correlation between training load and HR.^28^ Pregnant women with heart disease (valve disease or congenital heart disease) undergo the CPET at a submaximal level in specific situations, after excluding absolute contraindications. It is not recommended as a routine exam.

Analyzing the physical fitness of pregnant women can help identify those who have developed or are at risk of developing cardiovascular complications, such as systemic arterial hypertension or preeclampsia. The 6-minute walk test (6MWT) is a validated assessment of cardiorespiratory reserve in high-risk adults and non-pregnant women (for perioperative evaluation or chronic pathologies). Although it is submaximal, safe, viable and applicable in pregnant women at term, its use is not widespread. Since 6MWT reference ranges have been established for healthy pregnant women, individual assessment of functional capacity is possible, which can help personalize exercise programs.^29^^,^^30^ The 6MWT is recommended for the cardiorespiratory assessment of pregnant women.

Therefore, when there are pre-existing diseases and clinical or obstetric contraindications related to exercise, more specific cardiological examinations are required before exercising during pregnancy.

#### 2.3.1. Risk Stratification for Pregnant Women with Heart Disease

Patients with CVD should be stratified for exercise risk. The risk of complications in pregnancy depends on the underlying cardiac diagnosis, but an association of other comorbidities should also be considered. Therefore, risk estimates must be individualized.

Functional capacity and the clinical stability of heart disease are important factors when analyzing the risk of maternal complications, although it is often difficult to distinguish physiological signs and symptoms during pregnancy from those secondary to decompensated heart disease, such as edema, dyspnea, palpitation and dizziness. However, complaints such as palpitations, worsening functional capacity, nocturnal cough, orthopnea, paroxysmal nocturnal dyspnea, hemoptysis, precordial pain on exertion, or syncope should be followed up.^24^

In pregnant women with heart disease, a more detailed approach with specific exams is required, such as oxygen saturation, blood biochemistry (natriuretic peptide levels), echocardiogram (ventricular function, valve involvement, intrapulmonary pressure and aortic diameter), an exercise test or submaximal CPET (exercise capacity) and Holter monitoring (arrhythmias).^25^

The risk of heart disease during pregnancy can be stratified according to World Health Organization (WHO) parameters (Table 3).^31^^–^^33^ Women with heart disease are at increased risk for obstetric complications, including premature delivery, preeclampsia and postpartum hemorrhage. Fetal complications also occur in 18 to 30% of patients with heart disease, with neonatal mortality between 1 and 4%. Maternal and fetal events are highly correlated.^34^^,^^35^

**Table 3:** Modified World Health Organization classification system according to maternal cardiovascular risk

WHO modified	I	II	II-III	III	IV
Diagnostic	Small or light: – Pulmonary stenosis – Patent ductus arteriosus – Mitral valve prolapse (simple injuries successfully repaired) – Isolated atrial or ventricular ectopics	– Untreated atrioventricular septal defect – Corrected tetralogy of Fallot – Supraventricular arrhythmias – Turner's syndrome without aortic dilation	– Mild ventricular dysfunction (EF >45%) – Hypertrophic cardiomyopathy – Valve disease not considered WHO class I or IV (mild mitral stenosis, moderate aortic stenosis) – Marfan syndrome without aortic dilation – Corrected coarctation – Atrioventricular septal defect	– Moderate ventricular dysfunction (EF 30% to 45%) – Previous peripartum cardiomyopathy without residual ventricular dysfunction – Mechanical valve – Fontan circulation – Uncorrected cyanotic heart disease – Other complex heart diseases – Moderate mitral stenosis – Asymptomatic severe aortic stenosis – Systemic right ventricle with preserved function or mild dysfunction – Moderate aortic dilation (40 to 45 mm in Marfan syndrome; 45 to 50 mm in bicuspid aortic valve; aorta index 20 to 25 mm/m² in Turner syndrome; <50 mm in tetralogy of Fallot – Ventricular tachycardia	-Pulmonary arterial hypertension – Severe ventricular dysfunction (EF <30% or NYHA class III-IV) – Peripartum cardiomyopathy with residual ventricular dysfunction – Severe mitral stenosis – Symptomatic severe aortic stenosis – Systemic right ventricle with moderate or severe dysfunction – Severe aortic dilation (>45 mm in Marfan's syndrome; >50 mm in bicuspid aortic valve; aorta index >25 mm/m² in Turner's syndrome; >50 mm in tetralogy of Fallot – Ehlers-Danlos severe (re) coarctation – Fontan operation with complications
**Maternal cardiac event rate**	2.5% to 5%	5.7% to 10.5%	10% to 19%	19% to 27%	40% to 100%
**Risk**	No increased maternal risk of death detected and no/slightly increased risk of morbidity	Slightly increased risk of maternal death or moderate increase in morbidity	Increased risk of maternal death or moderate to severe increase in morbidity	Significant increase in risk of maternal death or severe morbidity	Extreme risk of maternal death or severe morbidity

*Adapted from Regitz-Zagrosek et al.*^25^*EF: ejection fraction; NYHA: New York Heart Association; WHO: World Health Organization.*

WHO classifications III and IV contraindicate exercise during pregnancy, and the other classes must be assessed individually (Figure 2).

### 2.4. Exercise Prescription

Exercise is here conceptualized as programmed physical activity, which involves repetitive body movement, aiming to improve or maintain one or more components of physical capacity.^36^

As a general rule, exercise programs must consider the pregnant woman’s physiological condition and incorporate these basic components: frequency, intensity, type and time (FITT). Frequency indicates the number of sessions per week, time indicates the duration of session, the types include aerobic and/or resistance, and intensity refers to the work level, which can be classified as light, moderate or intense.^36^

#### 2.4.1. Exercise Types and their Prescription for Pregnant Women

**Aerobic exercise:** Aerobic exercise metabolizes O2 as the main source of energy and involves cardiorespiratory demand. It comprises the flexion and extension of large muscle groups in a rhythmic manner for a prolonged time. However, when performed at high intensity, it may involve predominantly anaerobic metabolism, requiring other energy substrates to maintain effort, and a consequent risk of greater accumulation of lactic acid. Aerobic capacity, also known as VO2, reflects the cardiovascular system’s ability to transport and deliver O2 to skeletal muscles, in addition to the muscles’ efficiency at extracting it from the bloodstream and using it as an energy source for exercise.36

Regular aerobic exercise helps maintain functional capacity, control weight and prevent GDM and gestational hypertension. During pregnancy, low impact aerobic exercise, such as walking, swimming, stationary cycling, or water aerobics, is considered safe.^4^ Exercise performed on the floor or in a “dry” environment is more difficult during pregnancy, causing greater metabolic demand, osteoarticular overload and balance difficulties, because they are related to body weight, which progressively increases. Thus, aquatic exercise is considered preferential during this period. The effects of and recommendations about aquatic exercise will be addressed separately.

Since one of the main responses to aerobic exercise is increased HR and respiratory rate, the intensity can be based on HR control, which, despite its limitations in pregnant women, is the closest index of functional capacity. Based on healthy populations in both sexes, several methods for defining the intensity of aerobic exercise have been proposed, and these can be applied during normal pregnancies, including appropriate adjustments due to overload during that period.^36^

For healthy pregnant women, moderate-intensity exercise is recommended, although it is important to point out that the selected intensity is the combined responsibility of the doctor and exercise professional or physical therapist and can be reduced in previously sedentary women or those with comorbidities.^37^

The intensity of the exercise can be determined by calculating the percentage of the age-predicted maximum heart rate (HRmax) or the heart rate reserve (HRR). The percentage of maximum VO2 values and predicted metabolic load (MET) can also be used (Table 4).^36^ However, to obtain the HRR and maximum VO2, it is necessary to perform a maximum exercise test, which is not recommended for pregnant women. There are easier and more practical methods of prescribing exercise, such as subjective perceived exertion with a Borg scale^38^ (Table 5) and the talk test, ie, an exercise intensity at which the individual can talk, but not sing (Table 6).^3^^,^^37^^,^^39^

**Table 4:** Aerobic exercise intensity levels for healthy individuals

Intensity	%VO_2_ max	%HRmax	%HRR	MET* (absolute)	Borg Scale
Very light	<37	<57	<30	<2	<9
Light	37-45	57-64	30-40	2-3.9	9-11
Moderate	46-64	65-76	40-60	4-6	12-13
Intense	65-91	76-96	60-90	6,1-8,8	14-17
Very intense	>91	>96	>90	>8.9	>17

*Adapted from the ACSM.*^36^

* *MET: metabolic unit, which for non-pregnant women is equivalent to oxygen consumption of 3.5 ml/kg*^-1^*.min*^-1^ *while resting in a supine/sitting position. In the presented suggestions, oxygen consumption (VO2) values should ideally be obtained by a direct cardiopulmonary test and not estimated by regression equations derived from conventional exercise testing protocols; %VO2max: percentage of maximal oxygen consumption; %HRmax: percentage of age-predicted maximum heart rate; %HRR: percentage of heart rate reserve; Borg scale: linear scale of perceived exertion, graded from 6 to 20.*

**Table 5:** Borg scale of perceived exertion (graded from 6 to 20)

Borg Scale	Exercise perception
6	Very easy
7	
8	
9	Easy
10	
11	Relatively easy
12	
13	Slightly tiring
14	
15	Tiring
16	
17	Very tiring
18	
19	Exhausting
20	

*Adapted from Borg.*^38^

**Table 6:** Alternative methods of prescribing aerobic exercise for pregnant women*

METHOD	DESCRIPTION
Perceived exertion (Borg scale)	Exercise with relatively easy or slightly tiring self-perceived exertion. Borg score of 12 or 13 recommended
Talk test	Exercising at an intensity where breathing is labored but controlled, so that a sentence can be completed without pauses
Percentage of HRmax	Exercise at 60%-80% of HRmax Target HR = % x HRmax HRmax = 208 - (0.7 × age) ** or 220 - age ***
Heart rate reserve (*Karvonen*)	Exercise at 45%-60% of HRR (HR peak - HR rest) Target HR = resting HR + % × (peak HR - resting HR)

*HR: heart rate; HRmax: maximum heart rate; HRR: heart rate reserve.*

* *Considering a moderate intensity*

** *Tanaka’s formula*

*** *Karvonen’s formula*.^36^

Previously active women should continue regular aerobic exercise for at least 30 minutes, 4 to 5 times a week. Sedentary women can begin, for example, with 15 minutes of aerobic exercise 3 times a week and gradually increase the time until reaching the recommended 150 minutes/week or 30 minutes/day.^37^

**a) Resistance exercise:** loads specific muscle groups with weight to increase strength over time. When joint movement is associated with muscle contractions, the exercise is considered dynamic, when it is not the exercise is considered static (isometric). Resistance exercise can be performed with free weights, on weight machines, with elastic bands and even with one’s own body weight.^36^ The exercises must be adjusted in each gestational stage, adapting to the woman’s anatomical changes. Resistance exercise increases muscle tone, strength and endurance, which facilitates adaptation to postural changes, reducing musculoskeletal pain and preventing possible falls during pregnancy. It has been shown that regular strength training results in up to 14% higher lumbar resistance in pregnant women.^40^

Examples of resistance exercise include weight training, functional training, Pilates and yoga.^4^ Gentle stretching and yoga also promote muscle relaxation and are particularly useful in improving posture and relieving lower back and pelvic pain, although they should be performed with caution due to greater ligament laxity during pregnancy and the consequent risk of injury.^3^^,^^4^^,^^39^ Pilates provides satisfactory results when preparing the abdomen and pelvic floor for labor, in addition to reducing the risk of urinary incontinence. Pelvic muscle training has been found to reduce the occurrence of urinary incontinence by up to 50% in the prenatal period and 35% in the postnatal period.^41^

The intensity of the resistance exercise is defined according to the percentage of 1RM that the individual can perform, which is reflected in the number of possible repetitions with a given load. Table 7 describes the intensity ranges for resistance exercise training.^36^

**Table 7:** Resistance exercise intensity levels

Intensity	1RM percentage*	Number of repetitions
Light	30% to 50%	15 to 20
Moderate	50% to 70%	10 to 15
Intense	70% to 85%	8 to 10

* *1RM: maximum repetition.*

Moderate-intensity resistance exercise is recommended for pregnant women three to five times a week, in sessions lasting 15 to 20 min, approximately two to three sets of 10 to 15 repetitions.^3^^,^^36^^,^^37^ The number of repetitions and the load must be adjusted according to the individual’s previous muscle conditioning, ie, approximately 10 repetitions at the highest intensity/load. It is preferable to work with large muscle groups, using elastic bands or machines with light weights rather than free weights because of the greater difficulty balancing due to changes in center of gravity. Very high loads and intense isometrics are not recommended, since concomitant performance of the Valsalva maneuver can result in increased intra-abdominal pressure and a consequent decrease in blood flow to the fetus, in addition to further overload of the pelvic floor, increased risk of prolapse, and anal and urinary incontinence.^19^^,^^22^

**b) General exercise recommendations for healthy pregnant women** include aerobic and moderate-intensity resistance exercise. It is important to remember that the time calculated for the training session should include a mild-intensity warm-up and cool-down period. Exercise prescription for pregnant women according to the FITT components is summarized in Table 8.

**Table 8:** Exercise prescription for healthy pregnant women according to FITT

FREQUENCY	INTENSITY	TYPE	TIME
3 to 5 days/week	Moderate – 60% to 80% HRmax or – % of other validated method (HRR, VO2) or – 12 to 13 Borg scale or – Corresponding to 4 - 6 MET	Aerobic (low impact)	20 to 30 minutes per session (goal - a total of 150 minutes/week) 10 to 15 minutes heating and cooling
3 to 5 days/week	Moderate 2 to 3 sets and 10 to 12 repetitions	Resistance	15 to 20 minutes 10 to 15 minutes of warm-up and cool-down

*HRmax: maximum heart rate; HRR: heart rate reserve; MET: metabolic unit; VO2: oxygen consumption.*

#### 2.4.2. Aquatic Exercise

Aquatic exercise has been shown to be beneficial and safe during pregnancy. The aquatic environment provides buoyancy, which facilitates movement, reduces body weight and osteoarticular overload, and provides a feeling of well-being while eliminating the risk of falls. It also facilitates heat dissipation and thermoregulation, which reduces the chance of hyperthermia, which is unwanted during exercise due to the risk of fetal malformation.^42^ Thus, aquatic exercise (represented mainly by water aerobics) is the exercise of choice in this period and can include aerobic and resistance components.

Hydrostatic pressure, the main favorable effect of immersion for pregnant women, is proportional to depth and acts uniformly on the body surface, redistributing fluids from extravascular to intravascular space, which leads to rapid expansion of plasma volume, stroke volume and CO, as well as increased uteroplacental blood flow and diuresis. A study found that uterine tonus and fetal HR do not change during immersion or after aquatic exercise, demonstrating that neither uteroplacental circulation nor the fetal energy substrate are impaired.^43^

From a metabolic point of view, hormonal changes (increased natriuretic factor and decreased antidiuretic hormone levels secondary to receptor stimulation) increase diuresis for up to 4 hours. This effect contributes to a reduction in peripheral edema and lower BP levels after exertion.^44^

In comparison to floor (ie, dry medium) exercises, it has been observed that maternal HR, BP and temperature increases are lower in water and should be adjusted to the training range (approximately 15 beats per minute less). Aerobic performance seems to be better because, with less body weight, relative oxygen consumption is higher.^45^^,^^46^

To achieve these benefits, the chest should remain immersed to the xiphoid process or just below, with a water temperature between 28 and 30°C (never exceeding 33.4°C) and the sessions should last approximately 45 min, especially for untrained women. Foot support should be provided for those who are not good swimmers.

It has been demonstrated that swimming is safe during pregnancy, provided the recommended volume and intensity limits are observed. Interestingly, a recent study suggested that swimming in cold water (approximately 20°C, without artificial heating) can benefit both the mother and the fetus, since it is associated with decreased stress and cortisol levels (which are increased at rest), as well as an increased pain threshold.^47^ It should be noted, however, that this study was conducted with northern European women, who are more accustomed to such conditions.

Although SCUBA diving appears to be safe for pregnant women, it is not for the fetus. During sudden decompression, the fetal pulmonary circulation cannot filter bubbles, leading to the risk of gas embolism and fetal malformations. Therefore, SCUBA diving should be avoided, although snorkeling is allowed.^48^

The FITT recommendations for aquatic exercises during pregnancy are summarized in Table 9.

**Table 9:** Aquatic exercise guidelines

FREQUENCY	INTENSITY	TYPE	TIME
3 to 5 days/week	Moderate (for aerobic and resistance components)	Aqua aerobics or swimming	Up to 45 minutes/session

*Water temperature between 28 and 30°C; immersion level near the xiphoid process*

### 2.5. Exercises for Special Populations

#### 2.5.1. Hypertensive Disorders in Pregnancy

Hypertensive disorders occur in up to 10% of pregnancies and are associated with increased cardiovascular risk throughout life.^49^ They involve risk factors similar to those of the general population (advanced maternal age, ethnicity, family history of hypertension and sedentary lifestyle). The disorders are classified in Table 10.

**Table 10:** Classification of hypertensive disorders in pregnancy^25^

Hypertensive Pregnancy Disorders	
Pre-existing hypertension	Precedes pregnancy or develops before the 20th week of pregnancy. Persists for more than 42 days after delivery. May be associated with proteinuria
Gestational hypertension	Develops after the 20th week of pregnancy and resolves up to 42 days after delivery
Preeclampsia	Gestational hypertension with significant proteinuria: >0.3 g/24 h and/or albumin-creatinine ratio >30 mg/mmol

Gestational hypertension affects 5 to 8% of pregnancies and is characterized by systolic BP >140 mmHg and/or diastolic BP >90 mmHg. BP should be measured in a sitting position or in the left lateral position. Preeclampsia is a multisystem disorder that also occurs in 5% to 8% of pregnancies, although it is more frequent in the first pregnancy, multiple pregnancies, hydatidiform mole, antiphospholipid syndrome, arterial hypertension, kidney disease, or pre-existing diabetes. It is frequently associated with fetal growth restriction due to placental insufficiency (25% of cases), being a common cause of prematurity (27%) and intrauterine fetal death (4%). The main and most effective treatment is childbirth.^25^

The literature shows that regular exercise improves cardiovascular health during pregnancy and can decrease the risk of developing hypertensive disorders during pregnancy by up to 30%.^37^^,^^49^^,^^50^ Regular physical activity prior to pregnancy is related to a lower occurrence of preeclampsia, with 22% and 35% reductions in relative risk for women with moderate and high physical activity levels, respectively.^51^ This risk is even lower with combined physical activity before and early in pregnancy. When assessing the dose-response effect of physical activity, 5 to 6 h per week reduces the risk of preeclampsia by up to 40%, but no further reduction has been reported with higher activity levels.^25^^,^^50^

According to Brazilian and international guidelines, exercise is contraindicated in pregnant women with uncontrolled preexisting hypertension or a suspected/confirmed diagnosis of preeclampsia, and there is a relative contraindication for gestational hypertension.^49^^,^^50^

Other than consensus among specialists, no BP values have been established as exercise limits during pregnancy. The Brazilian Cardiovascular Rehabilitation Guideline recommends that an exercise session should not be started when resting BP levels are above 160/100 mmHg and it should be stopped BP reaches 220/105 mmHg in individuals with heart disease or hypertension.^52^

Based on these concepts, some precautions should be respected:

1. For pregnant women with controlled BP (<140/90 mmHg), mild-intensity exercise is recommended in sedentary patients, although a moderate level is acceptable for physically active women. BP measurements should be performed before, during and after exercise, interrupting activities when values above 160/100 mmHg are reached. The Valsalva maneuver should be avoided throughout pregnancy.
  - First trimester: at this stage, a reduction in BP levels is common, and many women tend not to use any specific medication for it. Discontinuation of antihypertensive drugs should be avoided; however, dosage adjustments may be necessary when water intake is increased. This will assist in correct hydration and body temperature control while exercising. Exercise intensity must be reduced at times.
  - Second trimester: during this period, BP stabilizes and rises again, and further therapeutic adjustments may be necessary. This trimester is the most hemodynamically stable and allows for slight load increases for women with adequate weight and controlled BP levels. Supine exercises should be avoided after this stage.
  - Third trimester: the mechanical compression that the uterus exerts on the aorta and inferior vena cava increases dramatically, resulting in greater stasis and edema in the lower limbs. BP is likely to increase due to water retention. Increased monitoring of BP levels is necessary, and exercise intensity should decrease. Self-limitations should guide exercise intensity and adherence during in this period. We suggest maintaining pelvic musculature conditioning, stretching and low- or very low-intensity aerobic exercise, as long as blood pressure levels remain within acceptable limits (≤160/100 mmHg). If there is any unexpected increase in blood pressure, exercise should be stopped.

#### 2.5.2. Diabetes Mellitus

GDM has short- and long-term complications for both mother and baby. Early diagnosis is important so that interventions can be carried out to reduce the deleterious effects of hyperglycemia. A high frequency of maternal dystocia and birth trauma is observed, with a greater chance of developing gestational hypertension or preeclampsia. In the long run, about 50% of these women develop type 2 DM and are at increased risk of post-term pregnancy; the child is also more likely to develop metabolic syndrome in childhood and adulthood.^53^^–^^55^

Lifestyle changes, such as diet and exercise, are the basis for treating GDM and are important allies when associated with drug treatment. In contrast, studies show that sedentary behavior increases the risk of GDM.^53^^,^^56^

In GDM, studies have shown that targeted exercise reduces negative maternal and fetal outcomes. However, this has not been proven for pregnant women with pre-existing DM.^53^^,57,58^ Nevertheless, in both populations, low- to moderate-intensity resistance and aerobic exercise helps with glycemic control and reduces the need for insulin, in addition to the other previously reported physical and psychological benefits.^53^^,^^58^

Special attention should be paid to contraindications to exercise, such as pre-proliferative retinopathy, uncontrolled hyperglycemia, hypoglycemia unawareness, advanced peripheral neuropathy and dysautonomia. Thirty-minute sessions of light- to moderate-intensity aerobic and resistance exercise at least three times a week are recommended for pregnant women with DM.^57^^,^^59^^,^^60^

One of the greatest risks for diabetic patients is hypoglycemia, which can occur during or after exercise. Capillary blood glucose monitoring should be performed before and after exercise, especially in pregnant women who are beginning an exercise program and after adjustments to the therapeutic regimen. At the beginning of exercise, capillary glycemia should be between 100 and 200 mg/dL. At levels below 100 mg/dL, 15 to 30 g of fast-absorbing carbohydrates should be consumed, which should be repeated after 30 min. Exercises must not be performed on an empty stomach or for more than 3 h without food. In cases of hyperglycemia with capillary levels above 250 mg/dL, exercise is contraindicated due to the risk of complications such as diabetic ketoacidosis.^57^^,^^60^

Some glycemic control issues for patients with type I diabetes should be highlighted when HbA1c levels are less than 7.5%.^61^ Patients must not exercise at the peak of insulin action, and insulin should not be applied in areas that will be most required during physical exertion due to greater absorption at the site.^57^^,^^59^^,^^60^ Uncontrolled type I DM is an absolute contraindication to exercise.

Although pregnancy is a challenging period for women who develop diabetes, it is also a greater motivation for healthy lifestyle changes, which can persist after birth and help prevent the onset of type 2 DM.

#### 2.5.3. Obesity

Pregnancy is included in the list of risk factors for obesity, predisposing women to GDM, type 2 DM, systemic arterial hypertension, CVD and cancer. The children of obese mothers also have a higher incidence of obesity and its metabolic and cardiovascular implications. There is also evidence that obese or overweight women have lower rates of breastfeeding initiation and duration, which is disadvantageous for their children’s growth and development.^62^^–^^64^

Obesity increases the probability of prolonged labor, probably due to lower myometrial tone, post-term pregnancy, cesarean delivery, prolonged hospitalization and puerperal infections. Contributing factors to the higher frequency of cesarean delivery include cephalopelvic disproportion and dystocia due to an increase in soft tissue in the maternal pelvis.^65^ In vaginal deliveries, the higher prevalence of macrosomia contributes to shoulder dystocia, causing perineal lacerations and brachial plexus paralysis in the newborn.^64^

Because obesity or excessive weight gain are linked to increased maternal and fetal complications during pregnancy, childbirth and the postpartum period, adherence to a balanced diet and regular exercise (preferably supervised) is strongly recommended.^65^^–^^67^ The main goal during pregnancy is not weight loss, but appropriate weight gain, avoiding excess in the third trimester. Maternal weight is an independent risk factor for preeclampsia, which doubles with each increase of 5 to 7 kg/m^2^ in pre-gestational body mass index.^68^ Prenatal physical training reduces weight gain and the risk of GDM for overweight and obese pregnant women.^64^

Several studies have demonstrated the safety of moderate-intensity exercise throughout pregnancy when there are no medical or obstetric contraindications.^3^^,^^69^ In obese pregnant women, light- to moderate-intensity aerobic exercise is recommended when prescribed at 35% to 60% of the HRR, which will depend on their previous cardiorespiratory condition. The benefits of endurance training can also be seen in this group.^37^^,^^39^

A key challenge is controlling adherence to regular physical activity, even in supervised programs.^69^ In view of all the favorable effects for both mother and fetus, overweight or obese women should be encouraged to abandon a sedentary lifestyle. In the postpartum period, exercise and a healthy diet must be encouraged, not only to lose excess gestational weight, but to achieve more appropriate weight goals for future pregnancies.

#### 2.5.4. Athletes

Athletic training presupposes great volumes of high-intensity exercise. In addition to the limitations imposed by pregnancy, ethical issues make it difficult to conduct studies, which results in little robust evidence on the safety of high-intensity exercise during pregnancy. However, some pregnant women choose to continue this level of training, including participation in competitions.

Active women who continue to exercise during pregnancy have lower resting HR and greater stroke volume, with better tolerance for high-intensity exertion, including during the puerperium, which facilitates a return to pre-pregnancy conditions and perhaps even better performance.^70^ Such data are relevant for athletes who intend to continue their careers and resume high-level training in the short term.

On the other hand, it must be considered that there is a decrease in functional residual capacity, a reduction in hepatic glycogen and greater difficulty in adapting to anaerobic exercise, with greater plasma accumulation of lactic acid and a decrease in glucose availability, especially during prolonged exercise. Compared to sedentary women, active women and their babies have lower weight, although this is due to the decreased body fat percentage, which damages neither mother nor child.^71^

Training and endurance tests, such as marathons, should be avoided because there is a risk of maternal hyperthermia in exercise sessions over 60 min (which can cause malformations of the neural tube if it occurs between the 4th and 6th week) and fetal hypoglycemia (identified by a transient decrease in fetus reactivity).^72^ When exercising in hot and humid environments, careful attention should be paid to hydration and caloric intake, which should be adjusted for increased metabolic needs to maintain fetal homeostasis.

Sports that involve collision with other players (basketball, football) or objects (hockey, volleyball) and falls (jumping, riding, cycling, skiing) are also discouraged, since they can cause placental detachment and/or fetal hypoxia or slowdown due to direct trauma.^3^^,^^19^

Despite these considerations, some elite athletes have continued to train and compete during pregnancy, winning titles in sports such as tennis, beach volleyball, track and field, marathons, mountain climbing and skiing, without no reported maternal-fetal complications.^65^^,^^73^^,^^74^

Although little data is available in the literature, a Norwegian study found that a group of elite athletes who continued training at a high volume and intensity on the 17th week of pregnancy benefitted (with no increased risks) from returning to competition after childbirth compared with a group that trained with a lower workload, which suggests that well-trained women can maintain normal exercise intensity during pregnancy.^75^ Another study carried out an exercise test until exhaustion (HR >90% of the predicted maximum) in three groups of pregnant women: inactive, active and very active (athletes), reporting decreased uterine artery flow and mild and transient fetal bradycardia (<3 min) in a small number of women. However, the meaning of this finding is unknown, considering that there was no damage to the fetus before or after the test or a greater occurrence of complications.^76^

In any case, the data are still scarce and further studies on pregnant athletes who continue high-intensity training are needed, provided that ethical principles and maternal-fetal safety are maintained. Although some groups believe that exercise recommendations for pregnant athletes could be more liberal, there is insufficient data to state that high-intensity exercise is safe during pregnancy. It is up to the doctor to understand the psychological and financial impact that these conditions can cause on the athlete’s well-being and career, advising her about the risks of complications and even unwanted terminations or irreversible damage to the baby. A shared decision is the best decision.

#### 2.5.5. Heart Disease

The changes that occur during pregnancy are dynamic, like an exercise test, with cardiovascular and systemic changes that can compromise the stability of heart disease. The increase in stroke volume, HR, CO and uterine blood flow and reduction in hematocrit and cardiac reserve may be sufficient to cause hemodynamic instability, not only at the beginning, but also throughout the gestational period.^25^^,^^77^ Regular consultations with a cardiologist should assist with clinical control in these patients.

Although regular exercise is recommended for primary and secondary prevention of CVD, heart disease in pregnant women has not been widely documented. Studies with this patient profile are scarce, given the complexity of most CVDs, such as congenital heart disease, heart valve disease, and pulmonary hypertension, conditions that endanger the life of the mother and fetus. Exercise prescription in this population should be based mainly on assessing cardiovascular risk during pregnancy. Important predictors of maternal cardiovascular events have been demonstrated in the literature, such as New York Heart Association (NYHA) functional class III and IV, moderate to severe obstructive lesions of the left heart, previous cardiac events, reduced left ventricular ejection fraction (<40%), moderate to severe systemic atrioventricular valve regurgitation, moderate to severe pulmonary atrioventricular valve regurgitation, pulmonary arterial hypertension, use of cardiac medication prior to pregnancy, cyanosis (oxygen saturation <90%), smoking, mechanical valve prosthesis, and corrected or uncorrected cyanotic heart disease.^24^

An interesting study by Jastrow et al. of 227 women with heart disease (312 pregnancies) used a risk score called Cardiac Disease in Pregnancy (CARPREG), whose variables are described in Table 11, to follow-up and determine the association with maternal and fetal outcomes. In the evaluated group, maternal cardiac injuries were predominantly congenital (81.4%), with a low risk score (score = 0) in 66.3%, and an intermediate risk (score = 1) in 33.7%. Maternal cardiac events complicated 7.4% of the pregnancies, with pulmonary edema being the most frequent (3.8%). Intermediate scores were associated with a higher rate of maternal cardiac outcomes (19.0% × 1.4%, odds ratio [OR] = 15.6, 95% CI), with adverse events occurring in 27.5% of the neonates. The CARPREG risk index showed high sensitivity and negative predictive value for cardiac complications in pregnant women with heart disease.^78^ However, when this index was applied to a sample of patients with rheumatic heart disease, it overestimated the number of events in pregnant women classified as CARPREG 1 and >1, in addition to underestimating the risk in low-risk patients (CARPREG 0).^79^

**Table 11:** Cardiac Disease in Pregnancy risk index

Predictors of Cardiovascular Events	Score
Previous cardiac event (heart failure, transient ischemic attack, infarction before pregnancy or arrhythmia)	1
Baseline NYHA functional class >II or cyanosis	1
Left-sided obstructive heart disease (mitral valve area <2 cm^2^, aortic valve <1.5 cm^2^ or peak gradient of outflow tract >30 mmHg	1
Reduced systolic ventricular function (ejection fraction <40%)	1

*Adapted from Martins et al.*^79^*NYHA: New York Heart Association.*

There are other indexes for assessing maternal-fetal risk, the most accurate of which are currently used by the WHO, as described above. Exercise is proscribed for pregnant women in WHO risk categories III and IV.^25^ For other cases, a detailed heart disease assessment is suggested, including consideration of the patient’s cardiorespiratory condition and the physiological changes peculiar to each gestational period, to assess the risks and benefits of exercise for the mother and fetus. The interaction between cardiology and obstetrics is very important in evaluating these patients and prescribing their exercise.

The use of functional tests in this scenario, such as exercise tests and the CPET, is controversial. Submaximal tests have been suggested for specific situations, including valve disease and congenital heart disease.^25^

HR, BP and peripheral oxygen saturation (by pulse oximetry) can guide prescription of the type and intensity of exercise, which should be applied by specialized trained professionals. To ensure that exercise is stopped when necessary, special attention must be paid to the warning signs and symptoms: tiredness, dyspnea, low output signs, and others described above.

Pregnant women with CVD at risk for potential complications (described in Table 12) should preferably be accompanied by a multidisciplinary team consisting of maternal-fetal medicine, cardiology, cardiovascular surgery, anesthesiology, and neonatology professionals and treated at a tertiary care center.^80^

**Table 12:** Main complications of cardiovascular disease during pregnancy

Cardiovascular Condition	Potential Complications
Arterial hypertension	– Preeclampsia; HELLP syndrome*
Mitral stenosis	– Tachyarrhythmias with low output, pulmonary edema
Mitral insufficiency	– Arrhythmia, pulmonary edema
Aortic stenosis	– Low cardiac output with preload drop
Aortic insufficiency	– Generally well tolerated – Congestive heart failure
Pulmonary stenosis Pulmonary insufficiency	– Usually well tolerated, – Pulmonary edema if right heart failure
Tricuspid stenosis	– Well tolerated
Tricuspid insufficiency	– Well tolerated
Cardiomyopathy	– Arrhythmia; Congestive heart failure; pulmonary edema
Aortic aneurysm	– Aortic dissection, rupture

*Adapted from Adam et al.*^80^

* *HELLP: hemolysis, elevated liver enzymes and low platelets.*

In Brazil, rheumatic disease is the most common heart disease in pregnant women, affecting mainly the mitral and aortic valves.^24^ Other heart diseases have a low prevalence in this population, and their heterogeneous presentation means that guidelines must be individualized. ^24^^,^^25^^,^^52^

Physical activity for those with valve diseases will depend on the affected valve and degree of impairment. Valve stenosis, particularly on the left side, leads to a higher risk of maternal complications than valve regurgitation, in which CO, vasodilation and tachycardia due to pregnancy are not well tolerated.^81^ Cavity size, ventricular function, pulmonary BP, association with other heart diseases, atrial fibrillation, heart failure, thromboembolism and previous endocarditis should also be analyzed, which are considered prognostic factors. According to the valvular heart disease risk classification for pregnancy (Table 13), which the Department of Women’s Cardiology used in its position statement,^24^ it is recommended that intermediate- and high-risk patients should not be allowed to exercise due to the severity of the heart disease and the consequent risk for mother and fetus.

**Table 13:** Classification of the risk of heart valve disease during pregnancy

High Risk	Intermediate Risk	Acceptable Risk
Severe mitral stenosis	Biological prosthesis with moderate dysfunction	Discreet valve disease
Severe aortic stenosis	Severe pulmonary stenosis	BP without dysfunction
Stenotic/calcified biological prosthesis	Metallic prosthesis	Valvulopathy + normal LVEF
Malfunctioning metallic prosthesis	Mitral metallic prosthesis > metallic prosthesis aorta risk	Valvulopathy without unfavorable factors
Valvulopathy + PAP ≥50 mmHg	Aortic insufficiency + aortic diseases	
Aortic insufficiency + aortic diseases	Marfan syndrome (Daorta between 40 and 45 mm)	
Marfan Syndrome (Daorta >45 mm)	Bicuspid aortic valve (Daorta between 45 and 50 mm)	
Bicuspid aortic valve (Daorta >50 mm)	Need for anticoagulants	
Valvulopathy + LVEF <35%		

*Adapted from Avila et al.*^24^*Daorta: diameter of the ascending aorta; LVEF: left ventricular ejection fraction; PAP: pulmonary artery pressure. Valve area ≤1 cm*^2^*is considered severe aortic or mitral stenosis.*

In mild valve lesions, when unfavorable factors are absent and functional capacity is within the appropriate age range, light-intensity exercise is suggested. It is acceptable to maintain moderate-intensity exercise only in pregnant women who have mild valve regurgitation without hemodynamic repercussions, as in mitral and aortic insufficiencies, especially for patients who have previously exercised.

It is important to monitor symptoms, echocardiographic findings and the peculiarities of each gestational period. In the third trimester, due to the physiological cardiovascular and respiratory changes and the weight gain, exercise should be reevaluated due to the risk of premature birth and delayed intrauterine growth. It must be remembered that although that physical exercise is important for the mother, it may be harmful to the fetus.

### 2.6. Precautions for Exercise during Pregnancy and Stopping Criteria

It is essential to maintain a healthy lifestyle during pregnancy, including measures such as maintaining adequate nutrition and rest, staying physically active, avoiding alcohol consumption, unnecessary medications, and active or passive smoking.

Pregnant women should be aware of certain exercise-related issues to avoid complications that are more evident in certain periods of pregnancy. Attention to diet is important: the caloric expenditure of the exercise must be estimated and balanced with adequate intake, and the signs and symptoms of hypoglycemia must be monitored. Exercising in hot and humid environments must be avoided, especially during the first trimester. Adequate hydration must be maintained, including isotonic fluids before and after exercise.

Beginning at the 10th week, special attention should be paid to stretching and sudden changes in exercise performance, when relaxin levels and the risk of injuries increase.^19^ Supine exercises can result in decreased venous return and hypotension in 10% to 20% of all pregnant women, causing lipothymia or syncope, especially after the 20th week. There is insufficient evidence to prove or disprove the safety of this type of exercise.^82^ If these symptoms occur, the exercises should be performed in lateral decubitus or standing. Abdominal exercises should also be avoided due to the increased risk of abdominal diastasis.

There is no specific period for stopping exercise during pregnancy. Mild-intensity exercises should be encouraged at the end of the third trimester and the patient should be advised about the signs of labor^37^, since there is a natural decrease in physical activity during this stage. Exercise should be stopped and a specialist consulted if symptoms of discomfort appear, such as shortness of breath that does not resolve with rest, severe headaches, regular and painful uterine contractions, vaginal bleeding, continuous loss of amniotic fluid, faintness or persistent dizziness, chest pain, palpitations, visual disturbances, persistent nausea and vomiting, muscle weakness that affects balance, etc.^3^

### 2.7. Exercise Recommendations and Adaptations during the Coronavirus Pandemic

The Ministry of Health included pregnant and puerperal women in the highest risk group for unfavorable outcomes in COVID-19. There is no evidence that pregnancy increases susceptibility to infection with SARS-CoV-2/COVID 19.^83^ In a systematic review of 18 articles published from February 12 to April 4 2020, of 108 pregnant women infected with SARS-CoV-2, 68% had fever, 34% cough, and 12% dyspnea.^84^

Physiological adaptations to pregnancy can aggravate some COVID-19 symptoms. Diaphragm displacement, decreased total lung capacity and chest compliance contribute to a worse outcome in patients with COVID-19 pneumonia, including hypoxia and fetal impairment. In addition to respiratory changes during pregnancy, cardiovascular and hematological changes should also be pointed out, such as increased HR, lower systemic vascular resistance and a hypercoagulable state. These factors have greater implications for pregnant women with heart disease who are infected with COVID-19.^85^

Thus, pregnant women should take extra precautions when exercising during the pandemic. Home exercise routines are recommended to reduce the risk of contamination. Mild- to moderate-intensity exercise is generally safe in a healthy pregnant woman and, as a general rule, certainly helps modulate emotional stress in addition to all the above mentioned health benefits. For pregnant women, the general guidelines for home exercise consist of a place with adequate space, ventilation and temperature, light clothing and tennis shoes, as well as adequate diet and hydration. Some exercises can be performed with common household materials.

For previously active pregnant women, exercise routines are easier to maintain, since they already understand how to perform the exercises, and understand perceived exertion and the warning symptoms for cessation. For sedentary women, the importance of exercise should be explained at the first consultation and mild-intensity exercises should be prescribed initially.

Home exercise, especially for sedentary pregnant women or those with comorbidities, should be supervised whenever possible by an exercise professional or physical therapist, which during the pandemic can include distance classes and telemonitoring, as deemed appropriate. According to the professional’s determination (always based on the relative benefits and safety), the first assessment could be in person, provided that measures of asepsis are followed.^86^^,^^87^

Considering that the quarantine is being relaxed heterogeneously in Brazil, with some public spaces reopened, when exercising outdoors, pregnant women should seek out open places with reduced circulation of people to lower the risk of contamination. There is a consensus that masks should be worn outside the home in addition to social distancing, especially when walking on city streets or in residential compounds. A recent Dutch study suggested a distance of 4 to 5 m should be maintained between people who are walking, 10 m between people who are running or cycling slowly, and 20 m between those cycling quickly.^88^

In apartment building gyms, hours should be reserved for residents of the same apartment and the equipment should be cleaned with 70% alcohol prior to use. Currently, gyms in many cities are open with limited hours and follow recommended security protocols.

Pregnant women who have been affected by COVID-19 should only return to regular physical activity after medical and ECG reevaluation, due to the possibility of myocardial injury and arrhythmias secondary to infection.^89^ In those with a more severe clinical evolution, an obstetrician should perform a thorough maternal-fetal assessment, a cardiologist should assess any cardiovascular complications, and complementary exams, such as transthoracic echocardiography, dynamic electrocardiography (eg, Holter monitoring), etc., should be performed.

## 3. Postpartum Exercise

When a woman exercises during pregnancy, there is a higher likelihood that she will continue to do so during the postpartum period, which has both short- and long-term benefits on quality of life. Women habitually reduce exercise after childbirth, due to the difficulties involved in this period. A sedentary lifestyle contributes to overweight and obesity and affects the development of other comorbidities, such as DM and CVD.^90^ Approximately 25% of women retain >4 kg in gained weight 1 year after delivery.^91^ Although exercise in the postpartum period is important for weight reduction, adjuvant therapies should also be considered. Some systematic reviews have shown that interventions involving a dietary component had a greater effect on weight loss. More intensive dietary interventions and more structured activity programs (eg, that incorporate HR monitoring) have been associated with greater weight loss.^92^^–^^94^

Obstetricians should emphasize the importance of exercise during this stage, including the cardiorespiratory benefits, fatigue reduction, improved sleep quality, and reduced risk of depression.^95^^–^^96^ It should be pointed out that postpartum depression is a prevalent condition, affecting approximately 10 to 15% of women in the first year after birth.^97^ Women who exercise during this period report a greater sense of well-being and improved quality of life.^98^^,^^99^

The postpartum exercise routine should gradually return to normal as soon as it is safe, which depends on the type of delivery (vaginal or cesarean) and whether there were surgical complications. It is recommended that exercise begin again approximately 6 weeks after cesarean delivery and 4 weeks after vaginal delivery. Progression should be slower if there is discomfort or other relevant factors, such as anemia or wound infection. Patients who exercised regularly prior to delivery should reduce the intensity in the first few months postpartum and progress gradually.^95^^,^^99^

Breastfeeding women can perform mild- to moderate-intensity aerobic exercise without damaging milk production or the growth of the child. Ideally, exercise should be performed after breastfeeding to avoid the discomfort of engorged breasts and should include adequate support. Women at this stage must maintain adequate hydration, even with satisfactory diuresis volume, as well as ensure that caloric intake is at least 75% of what is consumed in exercise; weight loss should be limited to 450 g per week. The caloric expenditure of breastfeeding is estimated at approximately 600 kcal/day.^100^^,^^101^ High-intensity exercise can be associated with increased lactic acid in breast milk, which changes the taste of milk. If the mother notices that the baby is rejecting breast milk, she should reduce her exercise level.^102^

Resistance exercises are allowed, but care should be taken not to involve trunk flexion (traditional abdominal exercises), due to the risk of increased diastasis in the rectus abdominis muscles. Pilates, a suitable option for training deep abdominal muscles and the pelvic floor, can help return the woman’s body to its pre-gestational state. Compromised pelvic muscles can contribute to urinary and fecal incontinence, as well as sexual dysfunction. Pelvic muscle training should be initiated during pregnancy. Jumping exercises should be avoided in the postpartum period due to the fragility of the pelvic floor.^103^

Exercise provides benefits during pregnancy, the puerperium period, and the long term. Women who continue to exercise are less likely to retain the excess weight acquired during pregnancy, develop depression, or develop metabolic and cardiovascular complications.^104^ All health professionals who monitor pregnant women should encourage safe exercise.

### 3.1. Particularities of the Postpartum Period for Patients with Heart Disease

The postpartum period involves important characteristics, especially for women with heart disease, since, in the immediate postpartum period, a significant change in blood volume occurs, which can lead to clinical decompensation. After the expulsive period of labor, there is a sudden increase in venous return, which is due to autotransfusion of the uterine plexus, decompression of inferior vena cava flow and reduced capacity in the venous system. In addition, peripheral vascular resistance is increased by sustained contraction of the uterus, which occludes vessels in the maternal surface of the placenta. The continuous autotransfusion that occurs 24 to 72 h after delivery involves a high risk of pulmonary congestion for women with heart disease.^105^

In general, changes in maternal blood volume during labor, the expulsive period and the puerperium involve the following phases: (1) hemoconcentration during labor, which varies with the degree of uterine activity and maternal dehydration; (2) reduced blood volume, which occurs during and immediately after delivery in proportion to the amount of blood lost; (3) an immediate and transient increase in blood volume, which occurs after placental discharge and is attributed to the influx of fluid into the intravascular space due to uterine emptying; (4) a slight increase in blood volume between the second and third postpartum days secondary to a transient increase in aldosterone secretion; (5) a reduction in plasma volume one week after delivery (the maternal stroke volume may show a slight drop in this period, but normalizes in the short term).^105^

As is well known, exercise and a healthy postpartum diet promote weight loss, which can improve or prevent future obesity-related risks, such as DM and systemic arterial hypertension.^64^^,^^106^ However, there are no robust data in the literature about postpartum exercise in patients with heart disease or complications during pregnancy. Therefore, exercise prescription follows the guidelines for specific heart diseases, being adapted to minimize risk and maternal injuries and maximize benefits.^24^^,^^52^

The most frequent cardiac complications for women with heart disease during the intrapartum and puerperium include preeclampsia, heart failure, acute pulmonary edema, arrhythmias, thromboembolism, and aortic dissection. Obstetric complications mainly include bleeding and infections.^24^ For vaginal deliveries, the degree of perineal trauma should be assessed. After cesarean delivery, other outcomes should be analyzed, such as cardiorespiratory disorders, thromboembolism, pain and the need for analgesia, abnormal wound healing, neuropathy and incontinence.^107^ Exercise should not be prescribed in the postpartum period until cardiac disease or clinical and surgical complications are controlled and a risk-benefit assessment of exercise is performed, a minimum of 4 weeks after vaginal delivery and 4 to 6 weeks after cesarean section. As soon as it is clinically safe, the patient should begin walking and return to normal pre-pregnancy physical activity, which can reduce the comorbidities associated with a sedentary post-surgical lifestyle.^108^^–^^110^

Physical activity should be resumed at the obstetrician’s discretion. When there are cardiovascular diseases and/or complications, joint assessment with a cardiologist becomes important.^111^^,^^112^ Due to a lack of data on physical exercise in heart disease patients during the postpartum period, most of the guidelines consist of extrapolations from cardiovascular rehabilitation guidelines. Therefore, more studies are needed to provide individualized and accurate care for this specific population.
